# HLA‐DR genotypes in patients with primary Sjögren's syndrome in Taiwan

**DOI:** 10.1002/kjm2.12885

**Published:** 2024-08-08

**Authors:** Chang‐Yi Yen, Pin‐Yi Wang, Kuan‐Yu Chen, Chia‐Chun Tseng, Cheng‐Chin Wu, Tsan‐Teng Ou, Jeng‐Hsien Yen

**Affiliations:** ^1^ Division of Rheumatology, Department of Internal Medicine Kaohsiung Medical University Hospital Kaohsiung Taiwan; ^2^ Institute of Medical Informatics, College of Electrical Engineering and Computer Science National Cheng Kung University Tainan Taiwan; ^3^ Department of Internal Medicine Kaohsiung Municipal Ta‐Tung Hospital Kaohsiung Taiwan; ^4^ Graduate Institute of Clinical Medicine, College of Medicine Kaohsiung Medical University Kaohsiung Taiwan; ^5^ School of Medicine, College of Medicine Kaohsiung Medical University Kaohsiung Taiwan; ^6^ Institute of Biomedical Science National Sun Yat‐Sen University Kaohsiung Taiwan; ^7^ Department of Biomedical Science and Technology National Yang Ming Chiao Tung University Hsinchu Taiwan

**Keywords:** genotype, human leukocyte antigen, Sjögren's syndrome

## Abstract

Different human leukocyte antigen (HLA) genotypes have been known to be associated with the risk of development of Sjögren's syndrome in different populations, but this association has never been reported in Taiwan. We enrolled 1044 subjects (673 patients, 371 controls) and tested their HLA‐DR genotypes. We found an increased risk of Sjögren's syndrome in patients carrying HLA‐DR8. DR1 and DR14 were associated with increased risk of eye involvement (uveitis, scleritis or optic neuritis), while DR15 was associated with increased risk of interstitial lung disease. DR8 was associated with increased risk of formation of multiple antibodies: anti‐Ro, rheumatoid factor and antinuclear antibodies (ANA) reaching titer 1:80 or above. DR9 was associated with decreased risk of formation of anti‐La antibodies and increased risk of formation of antithyroglobulin antibodies. DR10 was associated with risk of formation of anticyclic citrullinated peptide (anti‐CCP) antibodies, and DR11 was associated with increased risk of formation of anti‐La antibodies. Oral ulcer was found to be negatively associated with anti‐Ro antibodies and with anti‐ENA antibodies. Skin lesions were associated with ANA antibody titer elevation to 1:80 or above. Malignancies of any kind were associated with the presence of cryoglobulin. Females were more likely to be diagnosed at a younger age than males. There was no statistically significant relationship between HLA‐DR genotype and age at disease diagnosis. In patients with Sjögren's syndrome in Taiwan, the presence of HLA‐DR8 appeared to be a risk factor. In addition, we found several associations between HLA‐DR genotype, clinical presentation, and autoantibody status among them.

## 1 INTRODUCTION

Sjögren's syndrome is a chronic, progressive autoimmune disease that manifests primarily as the destruction of the salivary and lacrimal glands by infiltrating lymphocytes.^1^ Sjögren's syndrome can also present with extraglandular manifestations, which include constitutional symptoms, arthritis, involvement of parenchymal organs such as the lungs and kidney, immune complex‐mediated diseases such as vasculitis and membranoproliferative glomerulonephritis, and, later in the disease course, lymphoma.^2^ Sjögren's syndrome can occur per se (primary Sjögren's syndrome, hereafter referred to simply as Sjögren's syndrome) or in association with another autoimmune disease (secondary Sjögren's syndrome). Overall, the prevalence of Sjögren's syndrome is estimated to range from 0.01% to 0.05%.^3^

The development of Sjögren's syndrome is affected by genetic, epigenetic and environmental factors that are not yet completely defined. Genetic factors affecting disease susceptibility include certain HLA genotypes, as well as various single nucleotide polymorphisms (SNPs); other genetic loci have been identified in genome‐wide association studies.^4^ Defective epigenetic mechanisms, including abnormal DNA demethylation and abnormal microRNA expression, have been implicated in Sjögren's syndrome.^5^ Proposed environmental factors affecting disease susceptibility include viruses, such as Epstein–Barr virus, cytomegalovirus,^6^ and retroviral elements,^7^ as well as declining estrogen levels,^8^ which may explain the predominance of Sjögren's syndrome in postmenopausal women.

Of the genetic factors affecting susceptibility to Sjögren's syndrome, the best‐documented is HLA. Variations in the HLA genotype are long known to be associated with the susceptibility to, and presentation of, various autoimmune diseases in different populations.^9^, ^10^, ^11^ Notably, Sjögren's syndrome is well‐known to be associated with HLA‐DR genotypes.^12^, ^13^ To the best of our knowledge, there have been no reported studies examining the association between the HLA‐DR genotype and the risk of Sjögren's syndrome in Taiwan. This study aims to investigate the three‐way association between HLA‐DR genotypes, clinical manifestations, and autoimmune serological statuses in Taiwanese patients with Sjögren's syndrome.

## 2 MATERIALS AND METHODS

We performed a review of 1044 subjects, including 673 patients with Sjögren's syndrome, comprising 630 females and 43 males enrolled from the Rheumatology clinic at Kaohsiung Medical University Hospital, and 371 healthy controls, to investigate the relationship between HLA‐DR genotypes, the risk of development of Sjögren's syndrome, the clinical manifestations of Sjögren's syndrome, and associated autoantibody serologies in Taiwanese patients.

The collection of subject data was performed in accordance with the WMA Declaration of Helsinki, with the approval of the Institutional Review Board at Kaohsiung Medical University Hospital. All patients met the 2016 ACR‐EULAR Classification Criteria for Primary Sjögren's Syndrome.^14^ HLA‐DR genotyping was performed with the HLA FluoGene DRDQ kit (Inno‐Train Diagnostik GmbH, Kronberg, Germany). The medical records of each patient were reviewed to identify the disease manifestations, including parotid gland swelling, arthritis, oral ulcer, vasculitis, neuropathy, lymphadenopathy, annular erythematous skin lesions (including cutaneous lupus erythematosus (CLE)), eye involvement (including uveitis, scleritis or optic neuritis), interstitial lung disease, and malignancy. The autoimmune serologic profile for each patient was reviewed, including the serologic statuses for anti‐Ro antibody, anti‐La antibody, rheumatoid factor (RF), anticyclic citrullinated peptide (anti‐CCP) antibody, antinuclear antibody (ANA) (expressed in titer), anti‐double‐stranded DNA (anti‐dsDNA) antibody, antiextractable nuclear antigen (anti‐ENA) antibody, antithyroglobulin (ATG) antibody, antimicrosomal (AMS; antithyroid peroxidase) antibody, and cryoglobulins, as well as serum concentrations of complement C3 and complement C4. The biological gender and age at disease diagnosis for each patient were also reviewed.

Statistical analyses were performed using R (R Foundation for Statistical Computing, Vienna, Austria). Power analyses were performed using G*Power (Heinrich Heine University Düsseldorf, Düsseldorf, Germany). Alpha level was set at 0.05. *p*‐values were corrected for multiple comparisons using the Bonferroni and Benjamini‐Hochberg methods. Chi‐squared tests were performed to evaluate the association between each HLA‐DR genotype and disease status, between each genotype and the clinical manifestations, between each genotype and autoimmunity‐related markers, and between autoimmunity‐related markers and clinical manifestations. Two‐tailed *t*‐tests were performed to compare the mean ages at disease diagnosis between patients with and without each HLA‐DR genotype.

## 3 RESULTS

We tabulated the clinical manifestations (Table 1; Figure S1) and serological findings (Table 2; Figure S1) for the patient group, based on gender. We found that female patients were more likely to be diagnosed at an earlier age but found no significant difference in the frequencies of clinical manifestations and serological findings between the genders. The prevalence of each HLA‐DR genotype in the patient and control groups were also tabulated (Table 3; Figure S2). We found that HLA‐DR8 was associated with the occurrence of Sjögren's syndrome (odds ratio (OR) = 1.603, 95% confidence interval (CI) = 1.187–2.165, corrected *p* = 0.026). We then further compared the frequencies of each clinical manifestation for each genotype in patients with Sjögren's syndrome and found several associations between genotype and clinical manifestation (Table 4; Figure S3). DR1 and DR14 were associated with eye involvement (OR = 26.583, 95% CI = 2.576–274.348, corrected *p* = 0.00047; OR = 9.354, 95% CI = 1.541–56.766, corrected *p* = 0.0384 for DR1 and DR14, respectively). DR15 was associated with interstitial lung disease (OR = 4.731, 95% CI = 1.563–14.324, corrected *p* = 0.0312), while DR13 trended towards association (OR = 5.321, 95% CI 1.399–20.232, corrected *p* = 0.076). DR4 trended towards association with skin lesions (OR = 2.759, 95% CI = 1.333–5.709, corrected p = 0.055). DR8 trended towards association with lymphoma, but the odds ratio could not be calculated (corrected *p* = 0.086), since all study subjects who were diagnosed with lymphoma also carried DR8.

**TABLE 1:** Clinical manifestations in Sjögren's syndrome patients.

Clinical manifestation	Female, *n* = 630 (%)	Male, *n* = 43 (%)	*p* ^a^
Average diagnosis age^b^	52.144 ± 13.646	58.113 ± 13.589	0.0077
Parotid gland swelling	14 (2.22)	1 (2.33)	1.000
Arthritis	226 (35.87)	10 (23.26)	0.172
Oral ulcer	74 (11.75)	2 (4.65)	0.270
Vasculitis	21 (3.33)	0 (0)	0.465
Neuropathy	20 (3.17)	2 (4.65)	0.892
Lymphadenopathy	10 (1.59)	0 (0)	0.878
Skin lesions	30 (4.76)	1 (2.33)	0.755
Eye involvement	5 (0.79)	0 (0)	1.000
Interstitial lung disease	12 (1.90)	1 (2.33)	1.000
Malignancy	53 (8.41)	3 (6.98)	1.000
Malignancy (lymphoma)	3 (0.48)	0 (0)	1.000
Malignancy (nonlymphoma)	50 (7.94)	3 (6.98)	1.000

^a^ *p*‐value for average age at diagnosis is that from *t*‐test, while *p*‐values for clinical manifestations are those from chi‐square tests.

^b^ Mean ± standard deviation.

**TABLE 2:** Autoantibody serology in Sjögren's syndrome patients.

Presence of autoantibody	Female, *n* = 630 (%)	Male, *n* = 43 (%)	*p* ^a^
Anti‐Ro	410 (65.08)	25 (58.14)	0.607
Anti‐La	152 (24.13)	7 (16.28)	0.371
Anti‐ENA	421 (66.83)	25 (58.14)	0.986
Rheumatoid factor	170 (26.98)	7 (16.28)	0.458
Anti‐CCP	15 (2.38)	0 (0)	0.675
ANA≧1:80	265 (42.06)	10 (23.26)	0.084
Anti‐dsDNA	30 (4.76)	2 (4.65)	1.000
Low C3	117 (18.57)	7 (16.28)	1.000
Low C4	71 (11.27)	5 (11.63)	0.815
ATG Ab	72 (11.43)	1 (2.33)	0.265
AMS Ab	103 (16.35)	5 (11.63)	1.000
Cryoglobulin	13 (2.06)	0 (0)	0.967

Abbreviations: ATG, antithyroglobulin antibody; AMS, antimicrosomal (antithyroid peroxidase) antibody.

^a^ *p*‐values calculated for chi‐square tests.

**TABLE 3:** HLA genotypes in Sjögren's syndrome patients versus controls.

HLA genotype	Sjögren's, *n* = 673 (%)	Control, *n* = 371 (%)	Odds ratio, Sjögren/control (95% CI)	*p* ^a^	Corrected *p* ^b^
DR1	7 (1.04)	3 (0.81)	1.289 (0.331–5.016)	0.713	0.927
DR4	175 (26.00)	98 (26.42)	0.979 (0.734–1.306)	0.885	0.929
DR7	16 (2.38)	14 (3.77)	0.621 (0.300–1.287)	0.196	0.510
DR8	199 (29.57)	77 (20.75)	1.603 (1.187–2.165)	0.0020	0.026
DR9	145 (21.55)	98 (26.42)	0.765 (0.570–1.028)	0.075	0.280
DR10	10 (1.49)	6 (1.62)	0.918 (0.331–2.545)	0.869	0.929
DR11	110 (16.34)	54 (15.56)	1.147 (0.805–1.633)	0.447	0.830
DR12	170 (25.26)	84 (22.64)	1.155 (0.856–1.557)	0.345	0.748
DR13	39 (5.79)	22 (5.93)	0.976 (0.569–1.672)	0.929	0.929
DR14	96 (14.26)	58 (15.63)	0.898 (0.630–1.279)	0.551	0.895
DR15	136 (20.21)	71 (19.14)	1.070 (0.777–1.473)	0.678	0.927
DR16	71 (10.55)	27 (7.28)	1.503 (0.946–2.386)	0.083	0.280
DR17	88 (13.08)	63 (16.98)	0.735 (0.517–1.045)	0.086	0.280

^a^ *p*‐values calculated for chi‐squared tests.

^b^ *p*‐values corrected using Bonferroni method.

**TABLE 4:** HLA genotypes and associated clinical manifestations in Sjögren's syndrome patients (condensed).^a^

HLA genotype	Associated clinical manifestation	Odds ratio (95% CI)^b^	*p* ^c^	Corrected *p* ^d^
DR1	Eye involvement	26.583 (2.576–274.348)	0.000039	0.00047
DR4	Skin lesions	2.759 (1.333–5.709)	0.0046	0.055
DR8	Malig. (lymphoma)	Inf (NaN‐Inf)	0.0072	0.086
DR13	Interstitial lung disease	5.321 (1.399–20.232)	0.0063	0.076
DR14	Eye involvement	9.354 (1.541–56.766)	0.0032	0.0384
DR15	Interstitial lung disease	4.731 (1.563–14.324)	0.0026	0.0312

^a^ Only results with significant or near‐significant (i.e., between 0.05 and 0.10) corrected *p*‐values are shown here. See Table 4 in Supplementary information for the full table.

^b^ Some values could not be determined due to very low, very high, or lack of incidence. NaN, not a number; Inf: infinity.

^c^ *p*‐values calculated for chi‐square tests.

^d^ *p*‐values corrected with Benjamini‐Hochberg method.

We further examined the relationship between HLA‐DR genotype and autoantibody serology in patients (Table 5; Figure S4). DR8 was associated with anti‐Ro antibodies (OR = 2.169, 95% CI = 1.462–3.218, corrected *p* = 0.0012), RF (OR = 1.705, 95% CI = 1.170–2.485, corrected *p* = 0.0208), and the elevation of ANA titer above 1:80 (OR = 1.802, 95% CI = 1.243–2.611, corrected *p* = 0.0108). DR9 was associated with the presence of ATG antibodies (OR = 2.397, 95% CI = 1.389–4.138, corrected *p* = 0.0168), and negatively associated with anti‐La antibodies (OR = 0.480, 95% CI = 0.290–0.794, corrected *p* = 0.0222). DR10 was associated with anti‐CCP antibody (OR = 15.769, 95% CI = 2.646–93.971, corrected *p* = 0.00068). DR11 was associated with the presence of anti‐La antibodies (OR = 2.250, 95% CI = 1.448–3.498, corrected *p* = 0.0030). In addition, there was a trend towards negative association between DR4 and anti‐La antibodies (OR = 0.528, 95% CI 0.336–0.831, corrected *p* = 0.062), association between DR8 and anti‐La antibodies (OR = 1.582, 95% CI 1.083–2.311, corrected *p* = 0.051), and negative association between DR9 and RF (OR = 0.584, 95% CI 0.370–0.922, corrected *p* = 0.084).

**TABLE 5:** HLA genotypes and serology in Sjögren's syndrome patients (condensed).^a^

HLA genotype	Serology	Odds ratio (95% CI)^b^	*p* ^c^	Corrected *p* ^d^
DR4	Anti‐La	0.528 (0.336–0.831)	0.0052	0.062
DR8	Anti‐Ro	2.169 (1.462–3.218)	0.000096	0.0012
	Anti‐La	1.582 (1.083–2.311)	0.0171	0.051
	Rheumatoid factor	1.705 (1.170–2.485)	0.0052	0.0208
	ANA≧1:80	1.802 (1.243–2.611)	0.0018	0.0108
DR9	Anti‐La	0.480 (0.290–0.794)	0.0037	0.0222
	ATG Ab	2.397 (1.389–4.138)	0.0014	0.0168
DR10	Anti‐CCP	15.769 (2.646–93.971)	0.000062	0.00068
DR11	Anti‐La	2.250 (1.448–3.498)	0.00025	0.0030

^a^ Only results with significant or near‐significant (i.e., between 0.05 and 0.10) corrected p‐values are shown here. See Table 5 in Supplementary information for the full table.

^b^ Some values could not be determined due to very low incidence. NaN, not a number; Inf, infinity.

^c^ *p*‐values calculated for chi‐square tests.

^d^ *p*‐values corrected with Benjamini‐Hochberg method.

We also examined the relationship between serological status and clinical presentations in Sjögren syndrome patients (Table 6; Figure S5). Perhaps unsurprisingly, arthritis was positively associated with the presence of anti‐CCP antibodies (OR = 9.672, 95% CI 2.155–43.417, corrected *p* = 0.0041). Interestingly, oral ulcer was negatively associated with the presence of anti‐Ro antibodies (OR = 0.437, 95% CI 0.268–0.713, corrected *p* = 0.0043) and anti‐ENA antibodies (OR = 0.330, 95% CI 0.178–0.613, corrected *p* = 0.0032); in addition, there was a trend towards negative association with C4 hypocomplementemia (OR = 0.258, 95% CI 0.078–0.849, corrected *p* = 0.068). Skin lesions were associated with elevation of ANA titers to above 1:80 (OR = 3.552, 95% CI 1.548–8.150, corrected *p* = 0.0180). Nonlymphoma malignancies were associated with the presence of cryoglobulin (OR = 8.906, 95% CI 1.970–40.265, corrected *p* = 0.0168).

**TABLE 6:** Serologies and clinical manifestations in Sjögren syndrome patients (listed in order: odds ratio, 95% confidence interval, *p*‐value^a^, corrected *p*‐value^b^) (condensed).^c^

	Arthritis	Oral ulcer
Anti‐Ro	1.116 (0.790–1.577), 0.533, 0.914	0.437 (0.268–0.713), 0.00072, 0.0043
Anti‐ENA	0.817 (0.501–1.332), 0.418, 0.836	0.330 (0.178–0.613), 0.00027, 0.0032
Anti‐CCP	9.672 (2.155–43.417), 0.00034, 0.0041	1.048 (0.230–4.774), 0.952, 0.952

	Skin lesions	
ANA≧1:80	3.552 (1.548–8.150), 0.0015, 0.0180	

	Malignancy (total)	Malignancy (nonlymphoma)
Cryoglobulin	8.906 (1.970–40.265), 0.0014, 0.0168	8.906 (1.970–40.265), 0.0014, 0.0168

^a^ *p*‐values calculated for chi‐square tests.

^b^ *p*‐values corrected using Benjamini‐Hochberg method.

^c^ Only results with significant or near‐significant (i.e., between 0.05 and 0.10) corrected *p*‐values are shown here. See Table 6 in Supplementary information for the full table.

Finally, we compared the mean ages at disease diagnosis for each genotype in Sjögren's syndrome patients (Table 7; Figure S6). After correcting for multiple comparisons, we found no statistically significant association between HLA‐DR genotype and age at diagnosis. As we had found DR8 to be associated with an increased occurrence of Sjögren's syndrome, we performed a power analysis (post‐hoc 2‐tailed *z*‐test with an alpha of 0.05) to confirm whether we achieved adequate power to detect a difference in the proportion of DR8 in patients and controls and found that we achieved a power of 0.8805.

**TABLE 7:** HLA genotypes and mean age of diagnosis in Sjögren's syndrome patients.

HLA genotype	Mean age at diagnosis if positive (95% CI)	Mean age at diagnosis if negative (95% CI)	95% confidence interval for difference	*p* ^a^	Corrected *p* ^b^
DR1	53.932 (44.021–63.842)	52.525 (51.459–53.591)	−11.667, 8.853	0.750	0.949
DR4	52.246 (50.204–54.288)	52.644 (51.403–53.884)	−1.987, 2.782	0.743	0.949
DR7	51.857 (44.090–59.624)	52.555 (51.484–53.626)	−7.178, 8.574	0.852	0.949
DR8	50.967 (49.040–52.893)	53.217 (51.952–54.482)	−0.050, 4.551	0.055	0.358
DR9	52.434 (50.029–54.838)	52.569 (51.388–53.749)	−2.539, 2.809	0.921	0.949
DR10	51.073 (42.254–59.892)	52.563 (51.495–53.631)	−7.497, 10.477	0.718	0.949
DR11	52.442 (49.366–55.518)	52.559 (51.442–53.676)	−3.151, 3.386	0.944	0.949
DR12	51.609 (49.611–53.607)	52.843 (51.598–54.087)	−1.116, 3.583	0.302	0.785
DR13	50.474 (45.519–55.429)	52.669 (51.585–53.752)	−2.874, 7.262	0.387	0.839
DR14	54.868 (52.003–57.734)	52.150 (51.011–53.288)	−5.796, 0.359	0.083	0.360
DR15	53.672 (51.423–55.920)	52.248 (51.048–53.448)	−3.967, 1.120	0.271	0.785
DR16	52.437 (49.063–55.811)	52.552 (51.435–53.669)	−3.433, 3.663	0.949	0.949
DR17	55.608 (52.898–58.318)	52.077 (50.932–53.221)	−6.467, −0.595	0.019	0.247

^a^ *p*‐values calculated for two‐tailed t‐tests.

^b^ *p*‐values corrected using Benjamini‐Hochberg method.

## 4 DISCUSSION

In our study, we found that HLA‐DR8 was associated with the incidence of Sjögren's syndrome, as well as with the presence of anti‐Ro antibodies. In addition, we found several other associations between HLA‐DR genotype, autoantibody serology, and clinical manifestations.

A link between risk for Sjögren's syndrome and HLA serotype was first reported by Manthorpe et al.,^15^ in addition to a link between anti‐SSB/La and HLA serotype. Moriuchi et al.^16^ reported an increased frequency of HLA‐DRw53 (a supertype that includes HLA‐DR4, DR7, and DR9) in Japanese patients, while Kang et al.^12^ reported association between Sjögren's syndrome and HLA‐DR3 in Caucasian patients, HLA‐DR4 in Japanese patients, and HLA‐DR8 in Chinese patients.

Sjögren's syndrome is known to occur predominantly in women. In our study, in addition to confirming increased disease incidence in women, we found that the average age at which a patient was diagnosed with Sjögren's syndrome was significantly lower in women than in men, a finding that is compatible with previous epidemiological studies.^17^ It is currently unknown exactly why, among patients with Sjögren's syndrome, male patients seem to be diagnosed at older ages compared with female patients, although it has been proposed that since Sjögren's syndrome is much more prevalent in females than in males overall, there could be delayed recognition and diagnosis in male patients (interestingly, the US‐based Sjögren's Foundation further hypothesizes that the female‐to‐male ratio in Sjögren's syndrome could be reduced in the future, owing to increased clinical awareness of the disease in males).^18^ Additionally, it has also been proposed that males may be less likely to report or seek medical attention for early symptoms of Sjögren's syndrome, such as dry eyes and dry mouth, leading to under‐diagnosis. However, apart from this finding, we found neither any significant association between biological gender and clinical manifestation, nor any significant association between biological gender and autoantibody serological status. In contrast, Chatzis et al.^19^ reported an increased risk of lymphoma and an increased prevalence of serum anti‐La/SSB antibodies among male Sjögren's syndrome patients.

Sjögren's syndrome primarily involves exocrine glands, especially the lacrimal and salivary glands, but can involve other organ systems as well. For each HLA‐DR genotype, we investigated the relationship between the genotype and several clinical manifestations of Sjögren's syndrome (namely, parotid gland swelling, arthritis, oral ulcer, vasculitis, neuropathy, lymphadenopathy, skin lesions, eye involvement (uveitis, scleritis, or optic neuritis), interstitial lung disease, and malignancy).

Different HLA genotypes are known to be associated with different risks for various forms of uveitis. Zamecki and Jabs^20^ found that different HLA antigens were correlated with different presentations of uveitis. We found DR1 and DR14 to be associated with an increased risk of eye involvement in patients with Sjögren's syndrome.

Prior studies have established links between carriage of certain HLA genotypes and risk for interstitial lung disease. Xue et al.^21^ reported that DRB1*15:01 was over‐represented in patients with idiopathic pulmonary fibrosis, while Mori et al.^22^ reported that in patients with rheumatoid arthritis (RA) that DRB1*15:02 was positively associated with interstitial lung disease, but was negatively associated with airway disease. Furukawa et al.^23^ reported that, among Japanese patients with RA, the DRB1 shared epitope was negatively associated with risk for interstitial lung disease, while the DR2 serological group (DRB1*15 and *16) were positively associated. Fingerlin et al.^24^ performed a genome‐wide association study in non‐Hispanic White cases of fibrotic idiopathic interstitial pneumonia, and found that DRB1*15:01 and DQB1*06:02 were highly associated. The effects of HLA genotype on risk for pulmonary fibrosis are incompletely understood, but there appears to be an indirect association between the two. For example, in RA, there is an association between HLA epitopes and production of anti‐CCP antibodies, and an association between anti‐CCP antibodies and risk for pulmonary fibrosis; HLA and risk for pulmonary fibrosis are thus indirectly associated through anti‐CCP antibodies.^25^ In our study, we found that DR15 was significantly associated with interstitial lung disease in patients with Sjögren's syndrome; however, DR15 was not found to be associated with anti‐CCP antibody or anti‐Ro antibody, and anti‐CCP antibody and anti‐Ro antibody were not found to be associated with interstitial lung disease. It is possible that Sjögren's syndrome and RA cause interstitial lung disease via different pathogenic mechanisms; future work is needed to investigate these differences.

It is well‐established that certain HLA genotypes are correlated with risk for lymphoma.^26^ Among our subjects, we found no statistically significant correlation between malignancy (including lymphomas) and any of the HLA genotypes we tested for. DR8 trended towards association with increased risk of lymphoma. However, it should be noted that, of all our study subjects who simultaneously had Sjögren's syndrome and any form of malignancy, the most commonly occurring malignancy appeared to be solid organ cancers, rather than lymphomas. Our findings that no statistically significant link could be established between HLA genotype and malignancy could be due to the relatively low proportion of patients who simultaneously had Sjögren's syndrome and malignancy.

Different HLA genotypes are associated with different risks for SCLE and DLE in different populations. DLE is associated with HLA‐A28 in Black patients, and HLA‐B5 in White patients. However, the exact genotypes associated with each ethnic group can be controversial^27^, ^28^; on the other hand, it is less clear whether there is an association with Sjögren syndrome‐related skin lesions. We noted a trend towards association between DR4 and the presence of skin lesions, which includes CLE, although this association was not statistically significant.

No association was found between HLA‐DR genotype and the risks for parotid gland swelling, arthritis, and oral ulcer.

We examined the relationship between HLA‐DR genotype and various markers related to autoimmunity. Terao et al.^29^ reported that the SNP rs2395185 in the HLA locus, which is in linkage disequilibrium with HLA‐DRB1*0405, was significantly associated with ANA seropositivity. In our study, we found that HLA‐DR8 was significantly associated with an increase of ANA titer to 1:80 and above.

Anti‐CCP antibodies are highly specific for RA. van Drongelen and Holoshitz^30^ reviewed HLA disease associations in RA, and reported that patients carrying DR1, DR4, or DR10 (all of which encode a “shared epitope”) were more likely to carry anti‐CCP antibodies. We found in our study that anti‐CCP seropositivity was associated with DR10 but found no such association with DR1 or DR4 in Sjögren's syndrome.

Provost and Watson^31^ postulated that the association between HLA‐DR3 and Sjögren's syndrome is related to anti‐Ro/SSA. Miyagawa et al.^32^ reported in a study of Japanese women that a possible link exists between HLA class II genotype and the development of anti‐Ro and/or anti‐La antibodies. In our study, we found an association between the presence of anti‐Ro antibodies and DR8. This finding was consistent with those reported by Miyagawa et al. in Japanese subjects. In addition, we found a positive association between the presence of anti‐La antibodies and DR11, as well as a negative association with DR9.

Sjögren's syndrome is the most common rheumatic disease to be associated with autoimmune thyroid diseases, and HLA genotypes have long been known to be associated with thyroid disorders. The HLA haplotypes B8 and DR3 are frequently seen in both autoimmune thyroid disease and Sjögren's syndrome, and have been proposed to participate in both diseases.^33^ Thompson and Farid^34^ reported that Hashimoto's thyroiditis is associated with DR4, while Tachi et al.^35^ reported that patients with DRw9 and/or B51 and who had higher titers of ATG antibody were more likely to develop permanent hypothyroidism. Our results showed that DR9 was positively associated with the production of ATG antibodies. No similar associations were found for AMS antibodies.

Previous studies have investigated the association between HLA genotype and the presence of RF. Nelson et al.^36^ reported an association between DR4 and RF seropositivity in women with recent‐onset RA. We found DR8 to be significantly associated with the production of RF; DR9 trended towards negative association with RF.

We examined the relationship between various autoimmune markers and clinical manifestations of Sjögren's syndrome. We focus on anti‐Ro and anti‐La antibodies, which are frequently associated with Sjögren's syndrome. Anti‐Ro antibodies play a central role in the pathogenesis of Sjögren's syndrome. Over the past several decades, various associations have been reported between anti‐Ro/La serological status and the extraglandular manifestations of Sjögren's syndrome. Of these, an association between anti‐Ro/La serological status and skin manifestations, such as annular erythema, has been frequently reported.^37^ Initially, the association between anti‐Ro/La serological status and skin manifestations was believed to occur in patients of Asian descent. Brito‐Zerón et al.^38^ performed a retrospective study of non‐Asian primary Sjögren's syndrome patients who had presented with annular erythema, and found that annular erythema was strongly associated with anti‐Ro antibodies, but only weakly associated with other markers of immunity. However, in our study, no significant association between anti‐Ro or anti‐La with skin manifestations was detected.

In addition, we found that the presence of anti‐Ro antibodies was negatively associated with the incidence of oral ulcers. However, this finding remains unexplained, and to our knowledge, no mechanism for this finding has been proposed.

Finally, we examined the relationship between HLA‐DR genotype and age at disease diagnosis in Sjögren's syndrome patients. There is relatively scant information regarding the relationship between HLA genotype and age of onset in patients with Sjögren's syndrome. Thorlacius et al.^39^ found a correlation between HLA‐DQA1 and age at symptom onset. However, Cruz‐Tapias et al.^40^ reported no relationship between HLA and age at disease diagnosis in their review. In our study, we found no association between HLA genotype and the age at disease diagnosis.

In conclusion, HLA‐DR8 was found to be associated with a higher disease incidence and a higher percentage of anti‐Ro seropositivity. Several associations between HLA‐DR genotype, clinical presentation, and autoantibody status were uncovered. Further study is needed to explore the mechanistic link between HLA‐DR genotype and the overall clinical picture in Sjögren's syndrome in Taiwan.

## CONFLICT OF INTEREST STATEMENT

The authors declare no conflicts of interest.

## Supporting information


**Data S1.** Supporting information.

## References

[1] Hochberg MC , Gravallese EM , Smolen JS , Heijde DM van der , Weinblatt ME , Weisman MH , editors. Rheumatology. 8th ed. Philadelphia, PA: Elsevier; 2022.

[2] Loscalzo J , Fauci AS , Kasper DL , Hauser SL , Longo DL , Jameson JL , et al. Harrison's principles of internal medicine. 21st ed. New York, NY: McGraw Hill; 2022. p. 1.

[3] Beydon M , McCoy S , Nguyen Y , Sumida T , Mariette X , Seror R . Epidemiology of Sjögren syndrome. Nat Rev Rheumatol. 2024;20(3):158–169. 38110617 10.1038/s41584-023-01057-6

[4] Li Y , Zhang K , Chen H , Sun F , Xu J , Wu Z , et al. A genome‐wide association study in Han Chinese identifies a susceptibility locus for primary Sjögren's syndrome at 7q11.23. Nat Genet. 2013;45(11):1361–1365. 24097066 10.1038/ng.2779

[5] Konsta OD , Thabet Y , Le Dantec C , Brooks WH , Tzioufas AG , Pers JO , et al. The contribution of epigenetics in Sjögren's syndrome. The contribution of epigenetics in Sjögren's syndrome. Front Genet. 2014;5:71. 24765104 10.3389/fgene.2014.00071 PMC3982050

[6] Sorgato CC , Lins‐e‐Silva M , Leão JC , Vasconcelos LR , Romão TP , Duarte AL , et al. EBV and CMV viral load in rheumatoid arthritis and their role in associated Sjögren's syndrome. J Oral Pathol Med. 2020;49(7):693–700. 32428250 10.1111/jop.13036

[7] Yamano S , Renard JN , Mizuno F , Narita Y , Uchida Y , Higashiyama H , et al. Retrovirus in salivary glands from patients with Sjogren's syndrome. J Clin Pathol. 1997;50(3):223–230. 9155673 10.1136/jcp.50.3.223 PMC499817

[8] McCoy SS , Sampene E , Baer AN . Association of Sjögren's syndrome with reduced lifetime sex hormone exposure: a case‐control study. Arthritis Care Res. 2020;72(9):1315–1322. 10.1002/acr.24014 PMC6928446 31233285

[9] Dendrou CA , Petersen J , Rossjohn J , Fugger L . HLA variation and disease. Nat Rev Immunol. 2018;18(5):325–339. 29292391 10.1038/nri.2017.143

[10] Yen JH , Chen JR , Tsai WJ , Tsai JJ , Liu HW . HLA‐DRB1 genotyping in patients with rheumatoid arthritis in Taiwan. J Rheumatol. 1995;22(8):1450–1454. 7473464

[11] Yen CY , Wang PY , Chen KY , Tseng CC , Wu CC , Ou TT , et al. HLA‐DR genotypes in patients with systemic lupus erythematosus in Taiwan. J Chin Med Assoc. 2023;86(12):1060–1065. 37801591 10.1097/JCMA.0000000000001009 PMC12718894

[12] Kang HI , Fei HM , Saito I , Sawada S , Chen SL , Yi D , et al. Comparison of HLA class II genes in Caucasoid, Chinese, and Japanese patients with primary Sjögren's syndrome. J Immunol. 1993;150(8 Pt 1):3615–3623. 8468491

[13] Cobb BL , Lessard CJ , Harley JB , Moser KL . Genes and Sjögren's Syndrome. Rheum Dis Clin North Am. 2008;34(4):847–868. 18984408 10.1016/j.rdc.2008.08.003 PMC2994200

[14] Shiboski CH , Shiboski SC , Seror R , Criswell LA , Labetoulle M , Lietman TM , et al. 2016 American College of Rheumatology/European league against rheumatism classification criteria for primary Sjögren's syndrome: a consensus and data‐driven methodology involving three international patient cohorts. Arthritis Rheumatol. 2017;69(1):35–45. 27785888 10.1002/art.39859 PMC5650478

[15] Manthorpe R , Teppo AM , Bendixen G , Wegelius O . Antibodies to SS‐B in chronic inflammatory connective tissue diseases. Relationship with HLA‐Dw2 and HLA‐Dw3 antigens in primary Sjögren's syndrome. Arthritis Rheum. 1982;25(6):662–667. 6980008 10.1002/art.1780250609

[16] Moriuchi J , Ichikawa Y , Takaya M , Shimizu H , Uchiyama M , Sato K , et al. Familial Sjögren's syndrome in the Japanese: immunogenetic and serological studies. Clin Exp Rheumatol. 1986;4(3):237–241. 3769241

[17] Thurtle E , Grosjean A , Steenackers M , Strege K , Barcelos G , Goswami P . Epidemiology of Sjögren's: a systematic literature review. Rheumatol Ther. 2024;11(1):1–17. 37948031 10.1007/s40744-023-00611-8 PMC10796897

[18] Sjögren's Foundation [Internet] . Sjögren's in men. [cited 2024 Jul 16]. Available from: https://sjogrens.org/living-with-sjogrens/sjogrens-in-men

[19] Chatzis L , Pezoulas VC , Ferro F , Gandolfo S , Donati V , Binutti M , et al. Sjögren's syndrome: the clinical Spectrum of male patients. J Clin Med. 2020;9(8):2620. 32806710 10.3390/jcm9082620 PMC7463756

[20] Zamecki KJ , Jabs DA . HLA typing in uveitis: use and misuse. Am J Ophthalmol. 2010;149(2):189–193.e2. 20103052 10.1016/j.ajo.2009.09.018

[21] Xue J , Gochuico BR , Alawad AS , Feghali‐Bostwick CA , Noth I , Nathan SD , et al. The HLA class II allele DRB1*1501 is over‐represented in patients with idiopathic pulmonary fibrosis. PLoS ONE. 2011;6(2):e14715. 21373184 10.1371/journal.pone.0014715 PMC3044131

[22] Mori S , Koga Y , Sugimoto M . Different risk factors between interstitial lung disease and airway disease in rheumatoid arthritis. Respir Med. 2012;106(11):1591–1599. 22867979 10.1016/j.rmed.2012.07.006

[23] Furukawa H , Oka S , Shimada K , Sugii S , Ohashi J , Matsui T , et al. Association of Human Leukocyte Antigen with interstitial lung disease in rheumatoid arthritis: a protective role for shared epitope. PLoS ONE. 2012;7(5):e33133. 22586441 10.1371/journal.pone.0033133 PMC3346749

[24] Fingerlin TE , Zhang W , Yang IV , Ainsworth HC , Russell PH , Blumhagen RZ , et al. Genome‐wide imputation study identifies novel HLA locus for pulmonary fibrosis and potential role for auto‐immunity in fibrotic idiopathic interstitial pneumonia. BMC Genet. 2016;17(1):74. 27266705 10.1186/s12863-016-0377-2 PMC4895966

[25] Spagnolo P , Grunewald J , Du Bois RM . Genetic determinants of pulmonary fibrosis: evolving concepts. Lancet Respir Med. 2014;2(5):416–428. 24815806 10.1016/S2213-2600(14)70047-5

[26] Zhong C , Cozen W , Bolanos R , Song J , Wang SS . The role of hla variation in lymphoma aetiology and survival. J Intern Med. 2019;286(2):154–180. 31155783 10.1111/joim.12911

[27] Millard TP , Kondeatis E , Vaughan RW , Lewis CM , Khamashta MA , Hughes GR , et al. Polymorphic light eruption and the HLA DRB1*0301 extended haplotype are independent risk factors for cutaneous lupus erythematosus. Lupus. 2001;10(7):473–479. 11480844 10.1191/096120301678416024

[28] Fowler JF , Callen JP , Stelzer GT , Cotter PK . Human histocompatibility antigen associations in patients with chronic cutaneous lupus erythematosus. J Am Acad Dermatol. 1985;12(1 Pt 1):73–77. 3856581 10.1016/s0190-9622(85)70012-6

[29] Terao C , Ohmura K , Yamada R , Kawaguchi T , Shimizu M , Tabara Y , et al. Association between antinuclear antibodies and the HLA class II locus and heterogeneous characteristics of staining patterns: the Nagahama study. Arthritis Rheumatol. 2014;66(12):3395–3403. 25186300 10.1002/art.38867

[30] van Drongelen V , Holoshitz J . Human leukocyte antigen‐disease associations in rheumatoid arthritis. Rheum Dis Clin North Am. 2017;43(3):363–376. 28711139 10.1016/j.rdc.2017.04.003 PMC5643023

[31] Provost TT , Watson R . Anti‐Ro(SS‐A) HLA‐DR3‐positive women: the interrelationship between some ANA negative, SS, SCLE, and NLE mothers and SS/LE overlap female patients. J Invest Dermatol. 1993;100(1):14S–20S. 8423383 10.1111/1523-1747.ep12355186

[32] Miyagawa S , Shinohara K , Nakajima M , Kidoguchi KI , Fujita T , Fukumoto T , et al. Polymorphisms of HLA class II genes and autoimmune responses to Ro/SS‐A‐La/SS‐B among Japanese subjects. Arthritis Rheum. 1998;41(5):927–934. 9588746 10.1002/1529-0131(199805)41:5<927::AID-ART21>3.0.CO;2-R

[33] Robazzi TCMV , Adan LFF . Autoimmune thyroid disease in patients with rheumatic diseases. Rev Bras Reumatol. 2012;52(3):417–430. 22641595

[34] Thompson C , Farid NR . Post‐partum thyroiditis and goitrous (Hashimoto's) thyroiditis are associated with HLA‐DR4. Immunol Lett. 1985;11(5–6):301–303. 3879239 10.1016/0165-2478(85)90111-7

[35] Tachi J , Amino N , Tamaki H , Aozasa M , Iwatani Y , Miyai K . Long term follow‐up and HLA association in patients with postpartum hypothyroidism. J Clin Endocrinol Metab. 1988;66(3):480–484. 3162458 10.1210/jcem-66-3-480

[36] Nelson JL , Dugowson CE , Koepsell TD , Voigt LF , Branchaud AM , Barrington RA , et al. Rheumatoid factor, HLA‐DR4, and allelic variants of DRB1 in women with recent‐onset rheumatoid arthritis. Arthritis Rheum. 1994;37(5):673–680. 8185694 10.1002/art.1780370510

[37] Teramoto N , Katayama I , Arai H , Eto H , Kamimura K , Uetsuka M , et al. Annular erythema: a possible association with primary Sjögren's syndrome. J Am Acad Dermatol. 1989;20(4):596–601. 2715407 10.1016/s0190-9622(89)70070-0

[38] Brito‐Zerón P , Retamozo S , Akasbi M , Gandía M , Perez‐De‐Lis M , Soto‐Cardenas MJ , et al. Annular erythema in primary Sjogren's syndrome: description of 43 non‐Asian cases. Lupus. 2014;23(2):166–175. 24326481 10.1177/0961203313515764

[39] Thorlacius GE , Hultin‐Rosenberg L , Sandling JK , Bianchi M , Imgenberg‐Kreuz J , Pucholt P , et al. Genetic and clinical basis for two distinct subtypes of primary Sjögren's syndrome. Rheumatology. 2021;60(2):837–848. 32889544 10.1093/rheumatology/keaa367 PMC7850528

[40] Cruz‐Tapias P , Rojas‐Villarraga A , Maier‐Moore S , Anaya JM . HLA and Sjögren's syndrome susceptibility. A meta‐analysis of worldwide studies. Autoimmun Rev. 2012;11(4):281–287. 22001416 10.1016/j.autrev.2011.10.002

