# Supplementary material for: HLA‐DR genotypes in patients with primary Sjögren's syndrome in Taiwan
Source: Kaohsiung J Med Sci. 2024 Aug 8;40(10):934–41. doi: 10.1002/kjm2.12885 (PMC11895607; doi:10.1002/kjm2.12885)

HLA-DR Genotypes in Patients with Primary Sjögren’s Syndrome in Taiwan

Supplementary Materials

Table 4. HLA genotypes and associated clinical manifestations in Sjögren’s syndrome patients (full table)

| HLA genotype | Associated clinical manifestation | Odds ratio (95% CI) ^a^ | p-value ^b^ | Corrected p-value ^c^ |
| --- | --- | --- | --- | --- |
| DR1 | Parotid gland swelling | 0 (0-NaN) | 0.683 | 0.806 |
|  | Arthritis | 0.699 (0.135-3.632) | 0.669 | 0.806 |
|  | Oral ulcer | 3.081 (0.587-16.166) | 0.162 | 0.806 |
|  | Vasculitis | 0 (0-NaN) | 0.627 | 0.806 |
|  | Neuropathy | 0 (0-NaN) | 0.619 | 0.806 |
|  | Lymphadenopathy | 0 (0-NaN) | 0.739 | 0.806 |
|  | Skin lesions | 0 (0-NaN) | 0.552 | 0.806 |
|  | Eye involvement | 26.583 (2.576-274.348) | 0.000039 | 0.00047 |
|  | Interstitial lung disease | 0 (0-NaN) | 0.704 | 0.806 |
|  | Malignancy (total) | 1.8 (0.213-15.222) | 0.584 | 0.806 |
|  | Malig. (lymphoma) | 0 (0-NaN) | 0.857 | 0.857 |
|  | Malig. (nonlymphoma) | 1.913 (0.226-16.197) | 0.545 | 0.806 |
| DR4 | Parotid gland swelling | 1.019 (0.320-3.244) | 0.975 | 0.975 |
|  | Arthritis | 1.019 (0.710-1.463) | 0.919 | 0.975 |
|  | Oral ulcer | 1.241 (0.735-2.095) | 0.419 | 0.936 |
|  | Vasculitis | 2.149 (0.889-5.193) | 0.082 | 0.328 |
|  | Neuropathy | 2.388 (1.012-5.630) | 0.041 | 0.246 |
|  | Lymphadenopathy | 0.690 (0.145-3.280) | 0.639 | 0.935 |
|  | Skin lesions | 2.759 (1.333-5.709) | 0.0046 | 0.055 |
|  | Eye involvement | 0 (0-NaN) | 0.178 | 0.534 |
|  | Interstitial lung disease | 1.251 (0.380-4.116) | 0.712 | 0.935 |
|  | Malignancy (total) | 0.839 (0.439-1.600) | 0.593 | 0.935 |
|  | Malig. (lymphoma) | 1.409 (0.127-15.641) | 0.779 | 0.935 |
|  | Malig. (nonlymphoma) | 0.810 (0.415-1.581) | 0.537 | 0.935 |
| DR7 | Parotid gland swelling | 3.168 (0.389-25.790) | 0.255 | 0.510 |
|  | Arthritis | 0.431 (0.120-1.544) | 0.184 | 0.510 |
|  | Oral ulcer | 0.534 (0.069-4.121) | 0.541 | 0.791 |
|  | Vasculitis | 4.996 (1.053-23.707) | 0.025 | 0.300 |
|  | Neuropathy | 0 (0-NaN) | 0.464 | 0.791 |
|  | Lymphadenopathy | 0 (0-NaN) | 0.624 | 0.791 |
|  | Skin lesions | 1.440 0.183-11.320 | 0.727 | 0.791 |
|  | Eye involvement | 0 (0-NaN) | 0.730 | 0.791 |
|  | Interstitial lung disease | 4.000 0.484-33.080 | 0.165 | 0.510 |
|  | Malignancy (total) | 0 (0-NaN) | 0.231 | 0.510 |
|  | Malig. (lymphoma) | 0 (0-NaN) | 0.791 | 0.791 |
|  | Malig. (nonlymphoma) | 0 (0-NaN) | 0.245 | 0.510 |
| DR8 | Parotid gland swelling | 0.878 (0.276-2.792) | 0.825 | 0.825 |
|  | Arthritis | 0.898 (0.630-1.278) | 0.549 | 0.718 |
|  | Oral ulcer | 1.292 (0.778-2.146) | 0.321 | 0.718 |
|  | Vasculitis | 0.746 (0.269-2.066) | 0.572 | 0.718 |
|  | Neuropathy | 1.704 (0.716-4.056) | 0.223 | 0.718 |
|  | Lymphadenopathy | 1.624 (0.453-5.820) | 0.453 | 0.718 |
|  | Skin lesions | 0.829 (0.364-1.887) | 0.654 | 0.718 |
|  | Eye involvement | 0.602 (0.067-5.420) | 0.647 | 0.718 |
|  | Interstitial lung disease | 0.431 (0.095-1.963) | 0.263 | 0.718 |
|  | Malignancy (total) | 1.371 (0.772-2.435) | 0.280 | 0.718 |
|  | Malig. (lymphoma) | Inf (NaN-Inf) | 0.0072 | 0.086 |
|  | Malig. (nonlymphoma) | 1.146 (0.627-2.094) | 0.658 | 0.718 |
| DR9 | Parotid gland swelling | 0.884 (0.246-3.176) | 0.850 | 0.912 |
|  | Arthritis | 0.965 (0.655-1.421) | 0.856 | 0.912 |
|  | Oral ulcer | 1.212 (0.695-2.114) | 0.497 | 0.912 |
|  | Vasculitis | 0.831 (0.275-2.510) | 0.743 | 0.912 |
|  | Neuropathy | 0.785 (0.261-2.357) | 0.665 | 0.912 |
|  | Lymphadenopathy | 0.392 (0.049-3.124) | 0.360 | 0.912 |
|  | Skin lesions | 0.852 (0.343-2.120) | 0.731 | 0.912 |
|  | Eye involvement | 0.884 (0.098-7.970) | 0.912 | 0.912 |
|  | Interstitial lung disease | 0.290 (0.037-2.253) | 0.208 | 0.912 |
|  | Malignancy (total) | 0.763 (0.375-1.553) | 0.455 | 0.912 |
|  | Malig. (lymphoma) | 0 (0-NaN) | 0.359 | 0.912 |
|  | Malig. (nonlymphoma) | 0.822 (0.402-1.679) | 0.590 | 0.912 |
| DR10 | Parotid gland swelling | 0 (0-NaN) | 0.642 | 0.838 |
|  | Arthritis | 1.410 (0.375-5.304) | 0.609 | 0.838 |
|  | Oral ulcer | 2.193 (0.447-10.754) | 0.321 | 0.838 |
|  | Vasculitis | 0 (0-NaN) | 0.581 | 0.838 |
|  | Neuropathy | 0 (0-NaN) | 0.572 | 0.838 |
|  | Lymphadenopathy | 0 (0-NaN) | 0.705 | 0.838 |
|  | Skin lesions | 0 (0-NaN) | 0.499 | 0.838 |
|  | Eye involvement | 0 (0-NaN) | 0.790 | 0.838 |
|  | Interstitial lung disease | 0 (0-NaN) | 0.666 | 0.838 |
|  | Malignancy (total) | 0 (0-NaN) | 0.356 | 0.838 |
|  | Malig. (lymphoma) | 0 (0-NaN) | 0.838 | 0.838 |
|  | Malig. (nonlymphoma) | 0 (0-NaN) | 0.370 | 0.838 |
| DR11 | Parotid gland swelling | 1.900 (0.593-6.085) | 0.272 | 0.653 |
|  | Arthritis | 0.759 (0.486-1.187) | 0.226 | 0.653 |
|  | Oral ulcer | 0.959 (0.498-1.847) | 0.901 | 0.983 |
|  | Vasculitis | 0.532 (0.122-2.320) | 0.394 | 0.669 |
|  | Neuropathy | 1.522 (0.549-4.220) | 0.416 | 0.669 |
|  | Lymphadenopathy | 0.572 (0.072-4.560) | 0.593 | 0.791 |
|  | Skin lesions | 0.998 (0.374-2.661) | 0.997 | 0.997 |
|  | Eye involvement | 1.283 (0.142-11.597) | 0.824 | 0.983 |
|  | Interstitial lung disease | 0 (0-NaN) | 0.108 | 0.432 |
|  | Malignancy (total) | 0.273 (0.084-0.891) | 0.022 | 0.180 |
|  | Malig. (lymphoma) | 0 (0-NaN) | 0.446 | 0.669 |
|  | Malig. (nonlymphoma) | 0.291 (0.089-0.952) | 0.030 | 0.180 |
| DR12 | Parotid gland swelling | 1.509 (0.508-4.483) | 0.455 | 0.683 |
|  | Arthritis | 0.994 (0.687-1.437) | 0.973 | 0.973 |
|  | Oral ulcer | 0.847 (0.478-1.499) | 0.568 | 0.757 |
|  | Vasculitis | 0.490 (0.142-1.684) | 0.248 | 0.683 |
|  | Neuropathy | 0.658 (0.220-1.974) | 0.452 | 0.683 |
|  | Lymphadenopathy | 4.605 (1.283-16.528) | 0.010 | 0.120 |
|  | Skin lesions | 1.450 (0.668-3.148) | 0.345 | 0.683 |
|  | Eye involvement | 0.744 (0.083-6.703) | 0.791 | 0.863 |
|  | Interstitial lung disease | 0.538 (0.118-2.451) | 0.415 | 0.683 |
|  | Malignancy (total) | 1.582 (0.883-2.837) | 0.121 | 0.524 |
|  | Malig. (lymphoma) | 1.479 (0.133-16.414) | 0.749 | 0.863 |
|  | Malig. (nonlymphoma) | 1.581 (0.869-2.875) | 0.131 | 0.524 |
| DR13 | Parotid gland swelling | 1.188 (0.152-9.292) | 0.869 | 0.948 |
|  | Arthritis | 1.529 (0.785-2.981) | 0.209 | 0.484 |
|  | Oral ulcer | 0.200 (0.027-1.477) | 0.080 | 0.320 |
|  | Vasculitis | 0.825 (0.108-6.322) | 0.853 | 0.948 |
|  | Neuropathy | 0 (0-NaN) | 0.242 | 0.484 |
|  | Lymphadenopathy | 0 (0-NaN) | 0.433 | 0.742 |
|  | Skin lesions | 0 (0-NaN) | 0.161 | 0.483 |
|  | Eye involvement | 0 (0-NaN) | 0.581 | 0.813 |
|  | Interstitial lung disease | 5.321 (1.399-20.232) | 0.0063 | 0.076 |
|  | Malignancy (total) | 1.322 (0.451-3.876) | 0.610 | 0.813 |
|  | Malig. (lymphoma) | 8.569 (0.759-96.744) | 0.037 | 0.222 |
|  | Malig. (nonlymphoma) | 1.004 (0.298-3.385) | 0.995 | 0.995 |
| DR14 | Parotid gland swelling | 0 (0-NaN) | 0.109 | 0.436 |
|  | Arthritis | 1.464 (0.938-2.284) | 0.092 | 0.436 |
|  | Oral ulcer | 0.898 (0.444-1.817) | 0.765 | 0.893 |
|  | Vasculitis | 0.623 (0.143-2.722) | 0.526 | 0.789 |
|  | Neuropathy | 1.351 (0.447-4.084) | 0.593 | 0.791 |
|  | Lymphadenopathy | 2.614 (0.664-10.296) | 0.154 | 0.462 |
|  | Skin lesions | 0.882 (0.301-2.582) | 0.819 | 0.893 |
|  | Eye involvement | 9.354 (1.541-56.766) | 0.0032 | 0.0384 |
|  | Interstitial lung disease | 1.091 (0.238-5.002) | 0.911 | 0.911 |
|  | Malignancy (total) | 1.354 (0.658-2.788) | 0.409 | 0.789 |
|  | Malig. (lymphoma) | 0 (0-NaN) | 0.480 | 0.789 |
|  | Malig. (nonlymphoma) | 1.457 (0.705-3.011) | 0.307 | 0.737 |
| DR15 | Parotid gland swelling | 0.971 (0.270-3.492) | 0.964 | 0.988 |
|  | Arthritis | 1.003 (0.674-1.492) | 0.988 | 0.988 |
|  | Oral ulcer | 0.952 (0.523-1.735) | 0.873 | 0.988 |
|  | Vasculitis | 0.914 (0.302-2.762) | 0.873 | 0.988 |
|  | Neuropathy | 0.607 (0.177-2.084) | 0.423 | 0.846 |
|  | Lymphadenopathy | 0.431 (0.054-3.431) | 0.413 | 0.846 |
|  | Skin lesions | 0.930 (0.373-2.315) | 0.876 | 0.988 |
|  | Eye involvement | 0.979 (0.109-8.833) | 0.985 | 0.988 |
|  | Interstitial lung disease | 4.731 (1.563-14.324) | 0.0026 | 0.0312 |
|  | Malignancy (total) | 0.633 (0.292-1.373) | 0.244 | 0.846 |
|  | Malig. (lymphoma) | 0 (0-NaN) | 0.381 | 0.846 |
|  | Malig. (nonlymphoma) | 0.680 (0.312-1.479) | 0.328 | 0.846 |
| DR16 | Parotid gland swelling | 2.155 (0.593-7.834) | 0.233 | 0.875 |
|  | Arthritis | 1.068 (0.638-1.789) | 0.802 | 0.875 |
|  | Oral ulcer | 0.839 (0.369-1.908) | 0.676 | 0.875 |
|  | Vasculitis | 1.424 (0.409-4.964) | 0.577 | 0.875 |
|  | Neuropathy | 1.349 (0.389-4.682) | 0.636 | 0.875 |
|  | Lymphadenopathy | 0.933 (0.116-7.477) | 0.948 | 0.948 |
|  | Skin lesions | 1.263 (0.428-3.722) | 0.672 | 0.875 |
|  | Eye involvement | 0 (0-NaN) | 0.443 | 0.875 |
|  | Interstitial lung disease | 0.697 (0.089-5.446) | 0.730 | 0.875 |
|  | Malignancy (total) | 1.218 (0.529-2.803) | 0.643 | 0.875 |
|  | Malig. (lymphoma) | 0 (0-NaN) | 0.549 | 0.875 |
|  | Malig. (nonlymphoma) | 1.304 (0.565-3.012) | 0.533 | 0.875 |
| DR17 | Parotid gland swelling | 1.009 (0.224-4.551) | 0.991 | 0.991 |
|  | Arthritis | 0.745 (0.456-1.218) | 0.240 | 0.655 |
|  | Oral ulcer | 0.637 (0.283-1.436) | 0.273 | 0.655 |
|  | Vasculitis | 1.098 (0.317-3.810) | 0.882 | 0.962 |
|  | Neuropathy | 0 (0-NaN) | 0.063 | 0.378 |
|  | Lymphadenopathy | 0.724 (0.091-5.788) | 0.760 | 0.962 |
|  | Skin lesions | 0 (0-NaN) | 0.026 | 0.312 |
|  | Eye involvement | 1.644 (0.182-14.885) | 0.655 | 0.962 |
|  | Interstitial lung disease | 0 (0-NaN) | 0.155 | 0.620 |
|  | Malignancy (total) | 1.083 (0.494-2.374) | 0.841 | 0.962 |
|  | Malig. (lymphoma) | 0 (0-NaN) | 0.494 | 0.962 |
|  | Malig. (nonlymphoma) | 1.162 (0.529-2.556) | 0.708 | 0.962 |

^a^ Some values could not be determined due to very low, very high, or lack of incidence. NaN: not a number; Inf: infinity.

^b^ P-values calculated for chi-square tests.

^c^ P-values corrected with Benjamini-Hochberg method.

Table 5. HLA genotypes and serology in Sjögren’s syndrome patients (full table)

| HLA genotype | Serology | Odds ratio (95% CI) ^a^ | p-value ^b^ | Corrected p-value ^c^ | |
| --- | --- | --- | --- | --- | --- |
| DR1 | Anti-Ro | 0.358 (0.079-1.613) | 0.163 | 0.818 |  |
|  | Anti-La | 0.506 (0.060-4.238) | 0.522 | 0.818 |  |
|  | Anti-ENA | Inf (NaN-Inf) | 0.468 | 0.818 |  |
|  | Rheumatoid factor | 1.160 (0.211-6.392) | 0.865 | 0.952 |  |
|  | Anti-CCP | 0 (0-NaN) | 0.669 | 0.818 |  |
|  | ANA≧1:80 | 0.468 (0.104-2.108) | 0.311 | 0.818 |  |
|  | Anti-dsDNA | 0 (0-NaN) | 0.513 | 0.818 |  |
|  | Low C3 | 1.749 (0.289-10.592) | 0.538 | 0.818 |  |
|  | Low C4 | 0 (0-NaN) | 0.309 | 0.818 |  |
|  | ATG Ab ^d^ | 1.008 (0.116-8.760) | 0.994 | 0.994 |  |
|  | AMS Ab ^e^ | 0.613 (0.071-5.306) | 0.654 | 0.818 |  |
|  | Cryoglobulin | NaN (NaN-NaN) | NaN | NaN |  |
| DR4 | Anti-Ro | 0.797 (0.551-1.153) | 0.228 | 0.624 |  |
|  | Anti-La | 0.528 (0.336-0.831) | 0.0052 | 0.062 |  |
|  | Anti-ENA | 0.764 (0.449-1.302) | 0.321 | 0.624 |  |
|  | Rheumatoid factor | 0.828 (0.550-1.246) | 0.364 | 0.624 |  |
|  | Anti-CCP | 0.745 (0.206-2.692) | 0.653 | 0.784 |  |
|  | ANA≧1:80 | 0.983 (0.683-1.414) | 0.926 | 0.926 |  |
|  | Anti-dsDNA | 0.748 (0.314-1.781) | 0.510 | 0.765 |  |
|  | Low C3 | 1.278 (0.810-2.016) | 0.292 | 0.624 |  |
|  | Low C4 | 1.304 (0.763-2.229) | 0.331 | 0.624 |  |
|  | ATG Ab | 1.100 (0.625-1.936) | 0.741 | 0.808 |  |
|  | AMS Ab | 1.140 (0.700-1.858) | 0.599 | 0.784 |  |
|  | Cryoglobulin | 2.308 (0.645-8.253) | 0.191 | 0.624 |  |
| DR7 | Anti-Ro | 0.718 (0.252-2.045) | 0.534 | 0.783 |  |
|  | Anti-La | 1.545 (0.520-4.591) | 0.430 | 0.783 |  |
|  | Anti-ENA | Inf (NaN-Inf) | 0.206 | 0.783 |  |
|  | Rheumatoid factor | 1.162 (0.345-3.909) | 0.808 | 0.872 |  |
|  | Anti-CCP | 0 (0-NaN) | 0.587 | 0.783 |  |
|  | ANA≧1:80 | 2.566 (0.717-9.188) | 0.134 | 0.783 |  |
|  | Anti-dsDNA | 1.187 (0.147-9.579) | 0.872 | 0.872 |  |
|  | Low C3 | 0.572 (0.122-2.685) | 0.473 | 0.783 |  |
|  | Low C4 | 0.481 (0.061-3.817) | 0.479 | 0.783 |  |
|  | ATG Ab | 2.210 (0.558-8.755) | 0.247 | 0.783 |  |
|  | AMS Ab | 1.331 (0.338-5.238) | 0.682 | 0.818 |  |
|  | Cryoglobulin | 0 (0-NaN) | 0.414 | 0.783 |  |
| DR8 | Anti-Ro | 2.169 (1.462-3.218) | 0.000096 | 0.0012 |  |
|  | Anti-La | 1.582 (1.083-2.311) | 0.0171 | 0.051 |  |
|  | Anti-ENA | 1.175 (0.699-1.974) | 0.542 | 0.723 |  |
|  | Rheumatoid factor | 1.705 (1.170-2.485) | 0.0052 | 0.0208 |  |
|  | Anti-CCP | 0.578 (0.160-2.084) | 0.397 | 0.681 |  |
|  | ANA≧1:80 | 1.802 (1.243-2.611) | 0.0018 | 0.0108 |  |
|  | Anti-dsDNA | 1.537 (0.741-3.191) | 0.246 | 0.492 |  |
|  | Low C3 | 1.011 (0.651-1.571) | 0.960 | 0.960 |  |
|  | Low C4 | 1.074 (0.638-1.809) | 0.787 | 0.944 |  |
|  | ATG Ab | 1.217 (0.718-2.062) | 0.465 | 0.698 |  |
|  | AMS Ab | 0.970 (0.609-1.548) | 0.900 | 0.960 |  |
|  | Cryoglobulin | 2.218 (0.651-7.557) | 0.196 | 0.470 |  |
| DR9 | Anti-Ro | 0.665 (0.450-0.981) | 0.039 | 0.117 |  |
|  | Anti-La | 0.480 (0.290-0.794) | 0.0037 | 0.0222 |  |
|  | Anti-ENA | 0.819 (0.461-1.458) | 0.497 | 0.596 |  |
|  | Rheumatoid factor | 0.584 (0.370-0.922) | 0.0210 | 0.084 |  |
|  | Anti-CCP | 1.347 (0.419-4.333) | 0.616 | 0.672 |  |
|  | ANA≧1:80 | 0.730 (0.498-1.072) | 0.108 | 0.259 |  |
|  | Anti-dsDNA | 0.549 (0.187-1.613) | 0.269 | 0.514 |  |
|  | Low C3 | 0.935 (0.555-1.574) | 0.799 | 0.799 |  |
|  | Low C4 | 0.704 (0.362-1.370) | 0.300 | 0.514 |  |
|  | ATG Ab | 2.397 (1.389-4.138) | 0.0014 | 0.0168 |  |
|  | AMS Ab | 1.213 (0.725-2.030) | 0.461 | 0.596 |  |
|  | Cryoglobulin | 0.425 (0.050-3.647) | 0.423 | 0.596 |  |
| DR10 | Anti-Ro | 0.965 (0.239-3.897) | 0.960 | 0.964 |  |
|  | Anti-La | 2.483 (0.658-9.360) | 0.165 | 0.853 |  |
|  | Anti-ENA | 1.050 (0.125-8.843) | 0.964 | 0.964 |  |
|  | Rheumatoid factor | 1.397 (0.330-5.908) | 0.648 | 0.891 |  |
|  | Anti-CCP | 15.769 (2.646-93.971) | 0.000062 | 0.00068 |  |
|  | ANA≧1:80 | 0.625 (0.155-2.524) | 0.506 | 0.853 |  |
|  | Anti-dsDNA | 0 (0-NaN) | 0.406 | 0.853 |  |
|  | Low C3 | 0.866 (0.172-4.350) | 0.861 | 0.964 |  |
|  | Low C4 | 1.644 (0.325-8.305) | 0.543 | 0.853 |  |
|  | ATG Ab | 2.045 (0.389-10.750) | 0.388 | 0.853 |  |
|  | AMS Ab | 2.350 (0.518-10.670) | 0.255 | 0.853 |  |
|  | Cryoglobulin | NaN (NaN-NaN) | NaN | NaN |  |
| DR11 | Anti-Ro | 1.595 (0.990-2.569) | 0.054 | 0.162 |  |
|  | Anti-La | 2.250 (1.448-3.498) | 0.00025 | 0.0030 |  |
|  | Anti-ENA | 1.352 (0.684-2.672) | 0.385 | 0.490 |  |
|  | Rheumatoid factor | 1.387 (0.872-2.205) | 0.165 | 0.330 |  |
|  | Anti-CCP | 2.839 (0.938-8.587) | 0.054 | 0.162 |  |
|  | ANA≧1:80 | 1.291 (0.824-2.021) | 0.264 | 0.396 |  |
|  | Anti-dsDNA | 1.300 (0.540-3.129) | 0.558 | 0.558 |  |
|  | Low C3 | 0.792 (0.446-1.409) | 0.428 | 0.490 |  |
|  | Low C4 | 1.275 (0.680-2.390) | 0.449 | 0.490 |  |
|  | ATG Ab | 0.349 (0.135-0.901) | 0.0235 | 0.141 |  |
|  | AMS Ab | 0.605 (0.311-1.176) | 0.135 | 0.324 |  |
|  | Cryoglobulin | 0.280 (0.033-2.344) | 0.214 | 0.367 |  |
| DR12 | Anti-Ro | 0.966 (0.662-1.409) | 0.857 | 0.940 |  |
|  | Anti-La | 1.083 (0.720-1.629) | 0.702 | 0.940 |  |
|  | Anti-ENA | 1.284 (0.712-2.316) | 0.405 | 0.940 |  |
|  | Rheumatoid factor | 0.927 (0.614-1.400) | 0.719 | 0.940 |  |
|  | Anti-CCP | 0.851 (0.235-3.079) | 0.806 | 0.940 |  |
|  | ANA≧1:80 | 0.967 (0.666-1.406) | 0.862 | 0.940 |  |
|  | Anti-dsDNA | 0.738 (0.295-1.851) | 0.516 | 0.940 |  |
|  | Low C3 | 0.780 (0.473-1.285) | 0.328 | 0.940 |  |
|  | Low C4 | 0.809 (0.444-1.473) | 0.487 | 0.940 |  |
|  | ATG Ab | 0.608 (0.313-1.179) | 0.138 | 0.940 |  |
|  | AMS Ab | 1.001 (0.599-1.675) | 0.996 | 0.996 |  |
|  | Cryoglobulin | 2.098 (0.591-7.447) | 0.245 | 0.940 |  |
| DR13 | Anti-Ro | 0.746 (0.374-1.489) | 0.404 | 0.710 |  |
|  | Anti-La | 1.571 (0.767-3.220) | 0.214 | 0.710 |  |
|  | Anti-ENA | 0.552 (0.228-1.333) | 0.181 | 0.710 |  |
|  | Rheumatoid factor | 0.849 (0.402-1.795) | 0.669 | 0.859 |  |
|  | Anti-CCP | 0 (0-NaN) | 0.298 | 0.710 |  |
|  | ANA≧1:80 | 0.988 (0.495-1.971) | 0.973 | 0.973 |  |
|  | Anti-dsDNA | 0.685 (0.088-5.320) | 0.716 | 0.859 |  |
|  | Low C3 | 1.044 (0.396-2.755) | 0.931 | 0.973 |  |
|  | Low C4 | 1.563 (0.554-4.403) | 0.395 | 0.710 |  |
|  | ATG Ab | 0 (0-NaN) | 0.0169 | 0.203 |  |
|  | AMS Ab | 1.322 (0.562-3.112) | 0.522 | 0.783 |  |
|  | Cryoglobulin | 0 (0-NaN) | 0.414 | 0.710 |  |
| DR14 | Anti-Ro | 0.691 (0.439-1.086) | 0.108 | 0.296 |  |
|  | Anti-La | 0.545 (0.304-0.977) | 0.0393 | 0.236 |  |
|  | Anti-ENA | 0.656 (0.345-1.248) | 0.197 | 0.338 |  |
|  | Rheumatoid factor | 0.650 (0.377-1.122) | 0.120 | 0.296 |  |
|  | Anti-CCP | 0.890 (0.196-4.043) | 0.880 | 0.880 |  |
|  | ANA≧1:80 | 0.564 (0.361-0.883) | 0.0115 | 0.138 |  |
|  | Anti-dsDNA | 1.370 (0.502-3.739) | 0.537 | 0.806 |  |
|  | Low C3 | 0.896 (0.478-1.679) | 0.732 | 0.878 |  |
|  | Low C4 | 0.526 (0.217-1.272) | 0.148 | 0.296 |  |
|  | ATG Ab | 0.521 (0.215-1.260) | 0.142 | 0.296 |  |
|  | AMS Ab | 0.929 (0.489-1.766) | 0.823 | 0.880 |  |
|  | Cryoglobulin | 1.403 (0.256-7.677) | 0.695 | 0.878 |  |
| D15 | Anti-Ro | 1.265 (0.832-1.922) | 0.271 | 0.465 |  |
|  | Anti-La | 1.293 (0.843-1.983) | 0.239 | 0.465 |  |
|  | Anti-ENA | 1.610 (0.837-3.096) | 0.150 | 0.465 |  |
|  | Rheumatoid factor | 0.803 (0.515-1.253) | 0.333 | 0.488 |  |
|  | Anti-CCP | 0.982 (0.271-3.559) | 0.978 | 0.978 |  |
|  | ANA≧1:80 | 1.205 (0.804-1.807) | 0.366 | 0.488 |  |
|  | Anti-dsDNA | 0.549 (0.187-1.613) | 0.269 | 0.465 |  |
|  | Low C3 | 1.166 (0.711-1.914) | 0.542 | 0.591 |  |
|  | Low C4 | 0.796 (0.424-1.494) | 0.476 | 0.571 |  |
|  | ATG Ab | 1.418 (0.784-2.566) | 0.247 | 0.465 |  |
|  | AMS Ab | 1.414 (0.840-2.381) | 0.192 | 0.465 |  |
|  | Cryoglobulin | 0.234 (0.028-1.949) | 0.149 | 0.465 |  |
| DR16 | Anti-Ro | 1.247 (0.713-2.180) | 0.438 | 0.499 |  |
|  | Anti-La | 1.238 (0.704-2.177) | 0.457 | 0.499 |  |
|  | Anti-ENA | 1.481 (0.612-3.586) | 0.381 | 0.499 |  |
|  | Rheumatoid factor | 1.313 (0.755-2.282) | 0.333 | 0.499 |  |
|  | Anti-CCP | 0 (0-NaN) | 0.172 | 0.499 |  |
|  | ANA≧1:80 | 0.813 (0.484-1.368) | 0.436 | 0.499 |  |
|  | Anti-dsDNA | 0.563 (0.130-2.446) | 0.438 | 0.499 |  |
|  | Low C3 | 1.447 (0.759-2.759) | 0.260 | 0.499 |  |
|  | Low C4 | 0.435 (0.151-1.252) | 0.113 | 0.499 |  |
|  | ATG Ab | 0.586 (0.224-1.538) | 0.273 | 0.499 |  |
|  | AMS Ab | 0.621 (0.280-1.376) | 0.237 | 0.499 |  |
|  | Cryoglobulin | 1.403 (0.256-7.677) | 0.695 | 0.695 |  |
| DR17 | Anti-Ro | 0.818 (0.508-1.317) | 0.409 | 0.769 |  |
|  | Anti-La | 0.932 (0.545-1.594) | 0.797 | 0.956 |  |
|  | Anti-ENA | 0.656 (0.345-1.248) | 0.197 | 0.769 |  |
|  | Rheumatoid factor | 1.158 (0.692-1.937) | 0.577 | 0.769 |  |
|  | Anti-CCP | 0.413 (0.053-3.201) | 0.383 | 0.769 |  |
|  | ANA≧1:80 | 0.823 (0.514-1.318) | 0.418 | 0.769 |  |
|  | Anti-dsDNA | 1.031 (0.346-3.073) | 0.956 | 0.956 |  |
|  | Low C3 | 0.784 (0.405-1.516) | 0.468 | 0.769 |  |
|  | Low C4 | 2.035 (1.059-3.910) | 0.0304 | 0.365 |  |
|  | ATG Ab | 1.251 (0.629-2.487) | 0.522 | 0.769 |  |
|  | AMS Ab | 1.196 (0.652-2.191) | 0.563 | 0.769 |  |
|  | Cryoglobulin | 1.051 (0.199-5.550) | 0.954 | 0.956 |  |

^a^ Some values could not be determined due to very low incidence. NaN: not a number; Inf: infinity.

^b^ P-values calculated for chi-square tests.

^c^ P-values corrected with Benjamini-Hochberg method.

^d^ ATG: anti-thyroglobulin antibody

^e^ AMS: anti-microsomal (anti-thyroid peroxidase) antibody

Table 6. Serologies and clinical manifestations in Sjögren syndrome patients

(Listed in order: odds ratio, 95% confidence interval ^a^, p-value ^b^, corrected p-value ^c^) (full table)

|  | Parotid gland swelling | Arthritis | Oral ulcer |
| --- | --- | --- | --- |
| Anti-Ro | 0.774 (0.250-2.394), 0.655, 0.786 | 1.116 (0.790-1.577), 0.533, 0.914 | 0.437 (0.268-0.713), 0.00072, 0.0043 |
| Anti-La | 1.383 (0.420-4.553), 0.593, 0.786 | 1.043 (0.718-1.514), 0.826, 0.953 | 0.501 (0.257-0.977), 0.039, 0.103 |
| Anti-ENA | 0.340 (0.100-1.159), 0.071, 0.777 | 0.817 (0.501-1.332), 0.418, 0.836 | 0.330 (0.178-0.613), 0.00027, 0.0032 |
| RF | 2.076 (0.741-5.817), 0.156, 0.777 | 1.285 (0.897-1.841), 0.171, 0.684 | 0.535 (0.290-0.988), 0.043, 0.103 |
| Anti-CCP | 0 (0-NaN), 0.542, 0.786 | 9.672 (2.155-43.417), 0.00034, 0.0041 | 1.048 (0.230-4.774), 0.952, 0.952 |
| ANA≧1:80 | 1.949 (0.685-5.544), 0.203, 0.777 | 1.069 (0.770-1.485), 0.691, 0.953 | 0.602 (0.359-1.010), 0.053, 0.106 |
| dsDNA | 0 (0-NaN), 0.359, 0.786 | 1.980 (0.949-4.131), 0.065, 0.390 | 0.200 (0.027-1.497), 0.083, 0.125 |
| Hypo-C3 | 0.319 (0.039-2.578), 0.259, 0.777 | 1.033 (0.673-1.587), 0.881, 0.953 | 0.717 (0.364-1.412), 0.334, 0.401 |
| Hypo-C4 | 0.603 (0.074-4.896), 0.633, 0.786 | 1.015 (0.609-1.694), 0.953, 0.953 | 0.258 (0.078-0.849), 0.017, 0.068 |
| ATG Ab ^d^ | 1.530 (0.411-5.701), 0.523, 0.786 | 1.272 (0.762-2.124), 0.357, 0.836 | 1.517 (0.773-2.977), 0.224, 0.299 |
| AMS Ab ^e^ | 0.932 (0.252-3.450), 0.916, 0.916 | 1.307 (0.836-2.041), 0.240, 0.720 | 1.713 (0.950-3.089), 0.071, 0.122 |
| Cryoglobulin | 0.764 (0.084-6.944), 0.810, 0.884 | 1.190 (0.346-4.097), 0.782, 0.953 | 2.458 (0.206-29.338), 0.464, 0.506 |

|  | Vasculitis | Neuropathy | LAP |
| --- | --- | --- | --- |
| Anti-Ro | 2.105 (0.699-6.335), 0.176, 0.579 | 1.220 (0.466-3.191), 0.685, 0.783 | 0.478 (0.137-1.669), 0.237, 0.869 |
| Anti-La | 1.560 (0.618-3.936), 0.343, 0.579 | 1.560 (0.618-3.936), 0.343, 0.783 | 1.316 (0.336-5.152), 0.692, 0.869 |
| Anti-ENA | 2.879 (0.376-22.023), 0.287, 0.579 | 1.329 (0.298-5.928), 0.709, 0.783 | 0.428 (0.082-2.247), 0.302, 0.869 |
| RF | 1.727 (0.683-4.371), 0.243, 0.579 | 1.078 (0.403-2.883), 0.881, 0.881 | 1.162 (0.287-4.699), 0.833, 0.869 |
| Anti-CCP | 0 (0-NaN), 0.453, 0.604 | 0 (0-NaN), 0.453, 0.783 | 0 (0-NaN), 0.668, 0.869 |
| ANA≧1:80 | 1.164 (0.487-2.782), 0.733, 0.820 | 1.417 (0.593-3.389), 0.430, 0.783 | 0.545 (0.140-2.127), 0.375, 0.869 |
| dsDNA | 0 (0-NaN), 0.294, 0.579 | 1.560 (0.342-7.119), 0.563, 0.783 | 0 (0-NaN), 0.479, 0.869 |
| Hypo-C3 | 0.864 (0.273-2.731), 0.803, 0.820 | 0.736 (0.237-2.280), 0.593, 0.783 | 1.314 (0.238-7.267), 0.754, 0.869 |
| Hypo-C4 | 1.662 (0.521-5.299), 0.386, 0.579 | 0.600 (0.135-2.664), 0.497, 0.783 | 0 (0-NaN), 0.263, 0.869 |
| ATG Ab | 0 (0-NaN), 0.090, 0.579 | 1.391 (0.378-5.114), 0.618, 0.783 | 0.836 (0.099-7.046), 0.869, 0.869 |
| AMS Ab | 0.230 (0.030-1.779), 0.125, 0.579 | 1.242 (0.382-4.045), 0.718, 0.783 | 2.343 (0.516-10.637), 0.256, 0.869 |
| Cryoglobulin | 0.826 (0.160-4.269), 0.820, 0.820 | 5.364 (0.682-42.208), 0.080, 0.783 | 0 (0-NaN), 0.639, 0.869 |

|  | Skin lesions | Eye involvement | ILD |
| --- | --- | --- | --- |
| Anti-Ro | 1.409 (0.620-3.207), 0.411, 0.556 | 1.958 (0.217-17.626), 0.542, 0.745 | 2.717 (0.597-12.372), 0.179, 0.358 |
| Anti-La | 1.736 (0.813-3.708), 0.150, 0.450 | 0.769 (0.085-6.934), 0.815, 0.815 | 2.713 (0.898-8.198), 0.066, 0.358 |
| Anti-ENA | 4.343 (0.579-32.583), 0.120, 0.450 | Inf (NaN-Inf), 0.399, 0.688 | 0.968 (0.210-4.452), 0.966, 0.966 |
| RF | 1.389 (0.623-3.097), 0.420, 0.556 | 0 (0-NaN), 0.142, 0.688 | 2.376 (0.756-7.473), 0.127, 0.358 |
| Anti-CCP | 0 (0-NaN), 0.452, 0.556 | 0 (0-NaN), 0.740, 0.814 | 0 (0-NaN), 0.542, 0.650 |
| ANA≧1:80 | 3.552 (1.548-8.150), 0.0015, 0.0180 | 0 (0-NaN), 0.046, 0.506 | 2.604 (0.776-8.739), 0.108, 0.358 |
| dsDNA | 1.113 (0.249-4.969), 0.889, 0.889 | 0 (0-NaN), 0.618, 0.755 | 0 (0-NaN), 0.359, 0.615 |
| Hypo-C3 | 1.515 (0.651-3.525), 0.332, 0.556 | 0 (0-NaN), 0.283, 0.688 | 1.129 (0.287-4.436), 0.862, 0.940 |
| Hypo-C4 | 1.984 (0.798-4.930), 0.134, 0.450 | 0 (0-NaN), 0.430, 0.688 | 3.427 (0.943-12.456), 0.047, 0.358 |
| ATG Ab | 1.364 (0.440-4.235), 0.590, 0.644 | 0 (0-NaN), 0.438, 0.688 | 0 (0-NaN), 0.177, 0.358 |
| AMS Ab | 1.448 (0.537-3.907), 0.463, 0.556 | 0 (0-NaN), 0.322, 0.688 | 1.557 (0.383-6.335), 0.533, 0.650 |
| Cryoglobulin | 0 (0-NaN), 0.234, 0.556 | NaN (NaN-NaN), NaN, NaN | 0 (0-NaN), 0.414, 0.621 |

|  | Malignancy (total) | Malignancy (lymphoma) | Malignancy (nonlymphoma) |
| --- | --- | --- | --- |
| Anti-Ro | 1.280 (0.689-2.379), 0.434, 0.669 | Inf (NaN-Inf), 0.228, 0.787 | 1.173 (0.627-2.194), 0.617, 0.831 |
| Anti-La | 0.766 (0.385-1.523), 0.446, 0.669 | 6.178 (0.556-68.597), 0.091, 0.787 | 0.634 (0.302-1.334), 0.227, 0.454 |
| Anti-ENA | 1.150 (0.470-2.815), 0.760, 0.774 | Inf (NaN-Inf), 0.468, 0.787 | 1.054 (0.428-2.591), 0.909, 0.909 |
| RF | 1.705 (0.947-3.070), 0.072, 0.294 | 4.674 (0.421-51.886), 0.167, 0.787 | 1.580 (0.861-2.900), 0.137, 0.341 |
| Anti-CCP | 0 (0-NaN), 0.271, 0.542 | 0 (0-NaN), 0.787, 0.787 | 0 (0-NaN), 0.288, 0.494 |
| ANA≧1:80 | 1.088 (0.609-1.944), 0.774, 0.774 | 0.633 (0.057-7.020), 0.707, 0.787 | 1.127 (0.621-2.045), 0.694, 0.833 |
| dsDNA | 0.773 (0.176-3.394), 0.733, 0.774 | 0 (0-NaN), 0.680, 0.787 | 0.833 (0.189-3.669), 0.809, 0.883 |
| Hypo-C3 | 1.660 (0.825-3.341), 0.152, 0.365 | 0 (0-NaN), 0.380, 0.787 | 1.830 (0.899-3.725), 0.092, 0.341 |
| Hypo-C4 | 1.153 (0.487-2.732), 0.746, 0.774 | 0 (0-NaN), 0.521, 0.787 | 1.243 (0.522-2.959), 0.623, 0.831 |
| ATG Ab | 2.034 (0.864-4.788), 0.098, 0294 | 2.542 (0.227-28.404), 0.433, 0.787 | 1.948 (0.788-4.819), 0.142, 0.341 |
| AMS Ab | 1.985 (0.906-4.346), 0.082, 0.294 | 1.547 (0.139-17.227), 0.721, 0.787 | 2.022 (0.889-4.599), 0.088, 0.341 |
| Cryoglobulin | 8.906 (1.970-40.265), 0.0014, 0.0168 | NaN (NaN-NaN), NaN, NaN | 8.906 (1.970-40.265), 0.0014, 0.0168 |

^a^ Some values could not be determined due to very low, very high, or lack of incidence. NaN: not a number; Inf: infinity.

^b^ P-values calculated for chi-square tests.

^c^ P-values corrected using Benjamini-Hochberg method.

^d^ ATG: anti-thyroglobulin antibody

^e^ AMS: anti-microsomal (anti-thyroid peroxidase) antibody

Figure S1. Characteristics of patient group


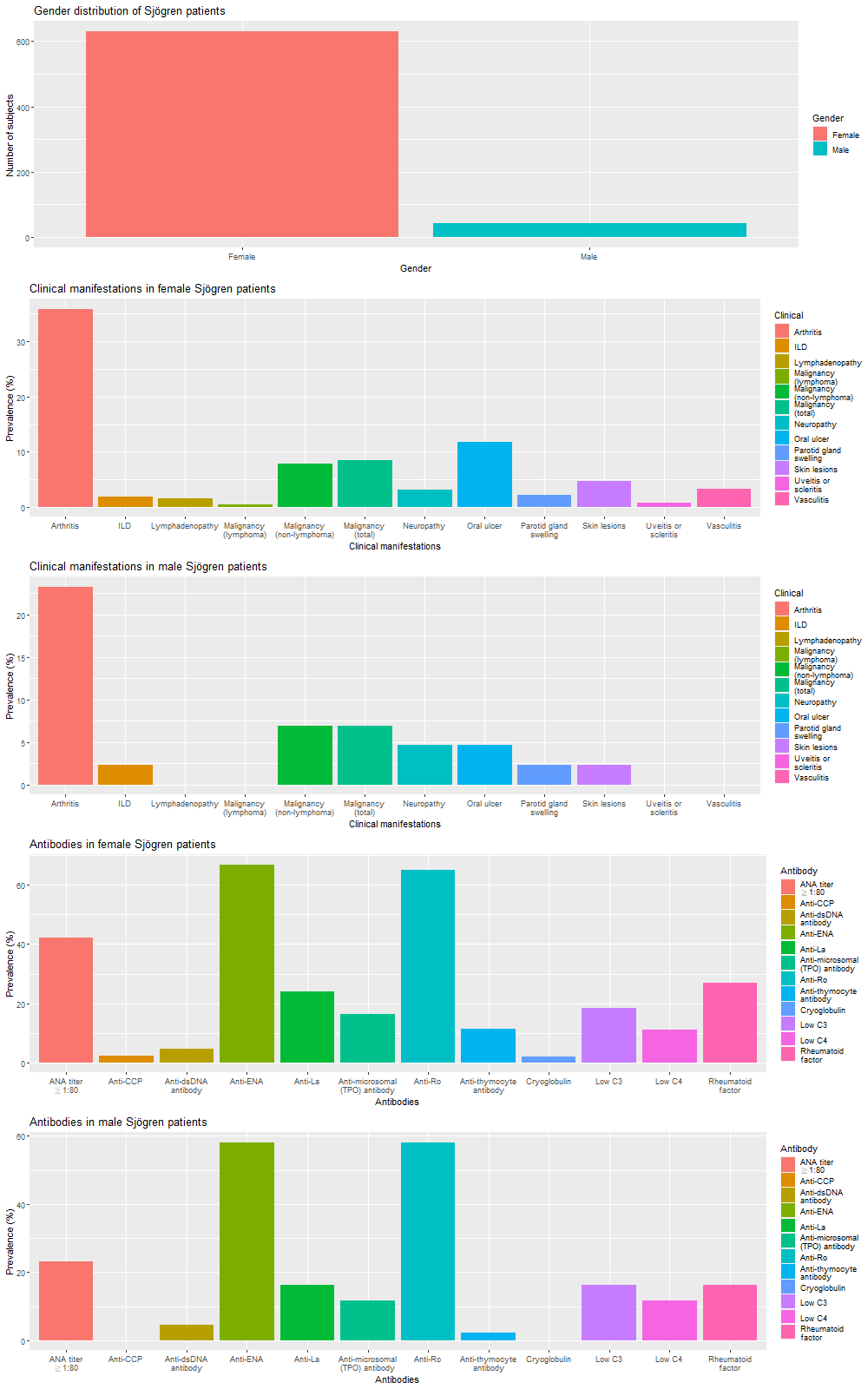


Figure S2. Prevalence of HLA-DR genotypes in patient and control groups


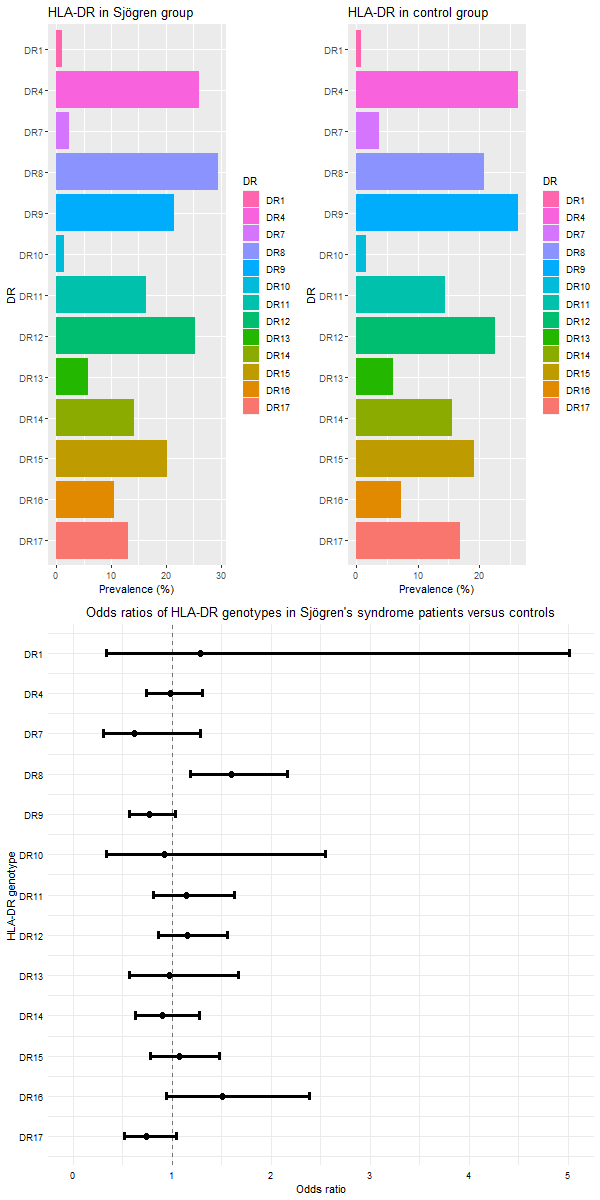


Figure S3. Log-odds ratios of clinical manifestations by HLA-DR genotype


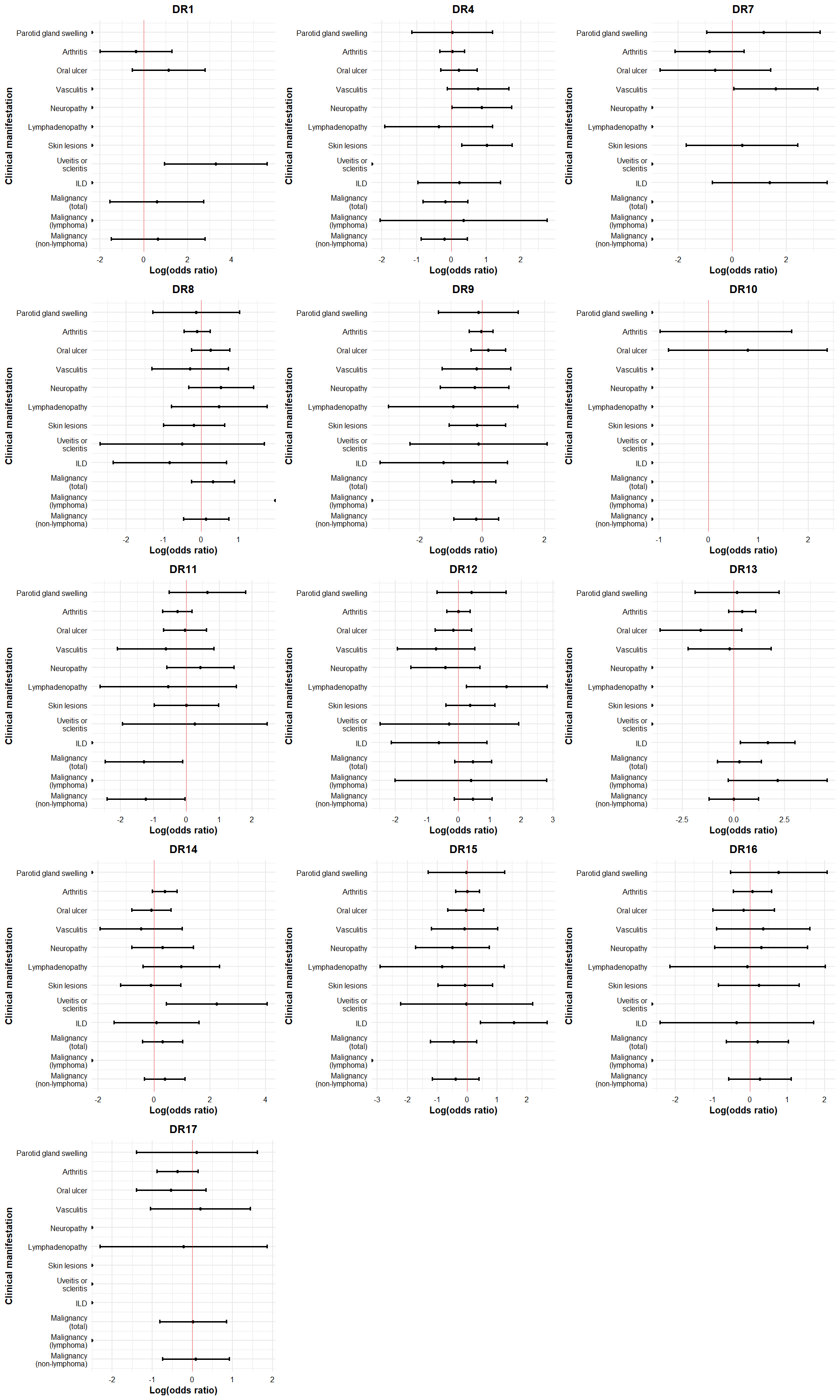


Figure S4. Log-odds ratios of serological findings by HLA-DR genotype


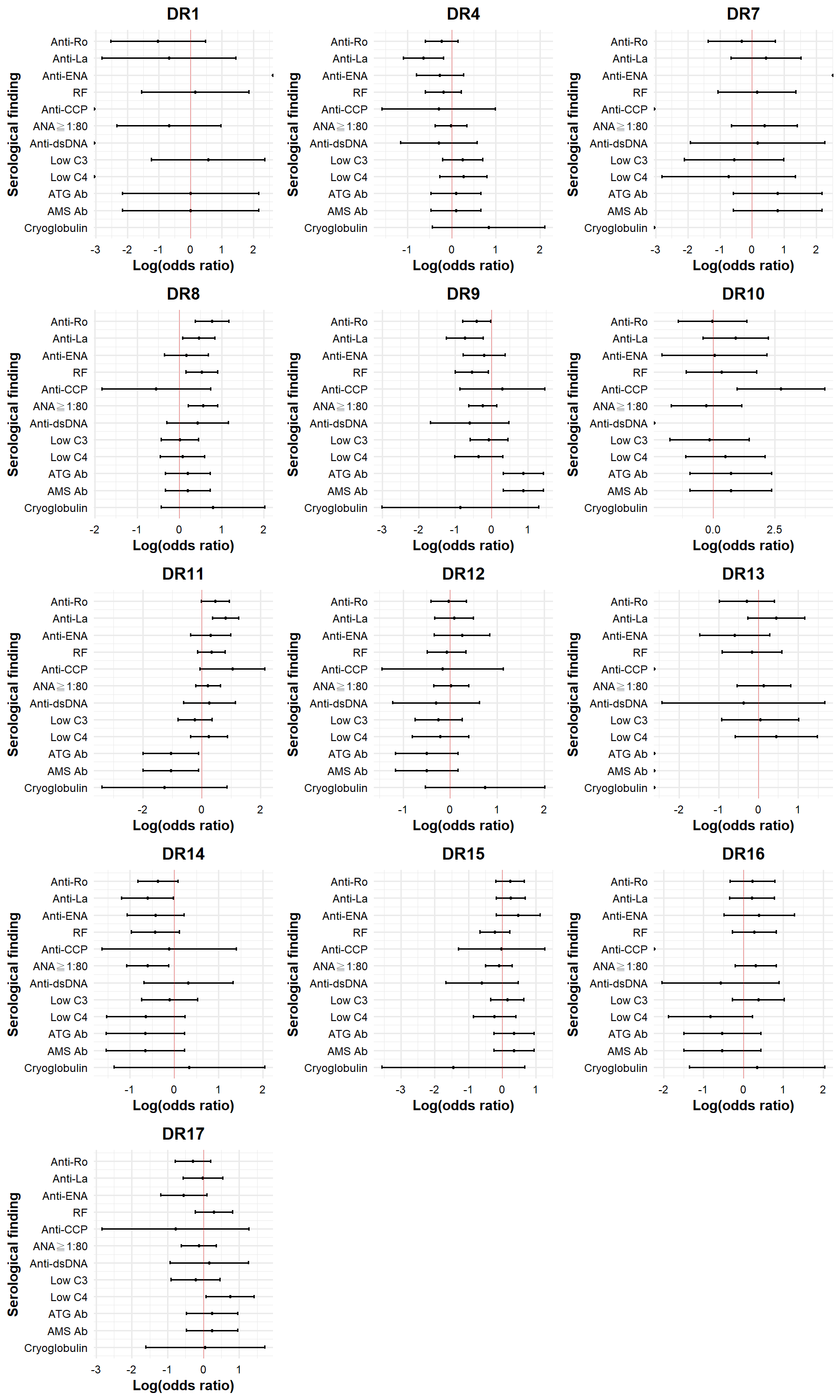


Figure S5. Log-odds ratios of serological findings by clinical presentation


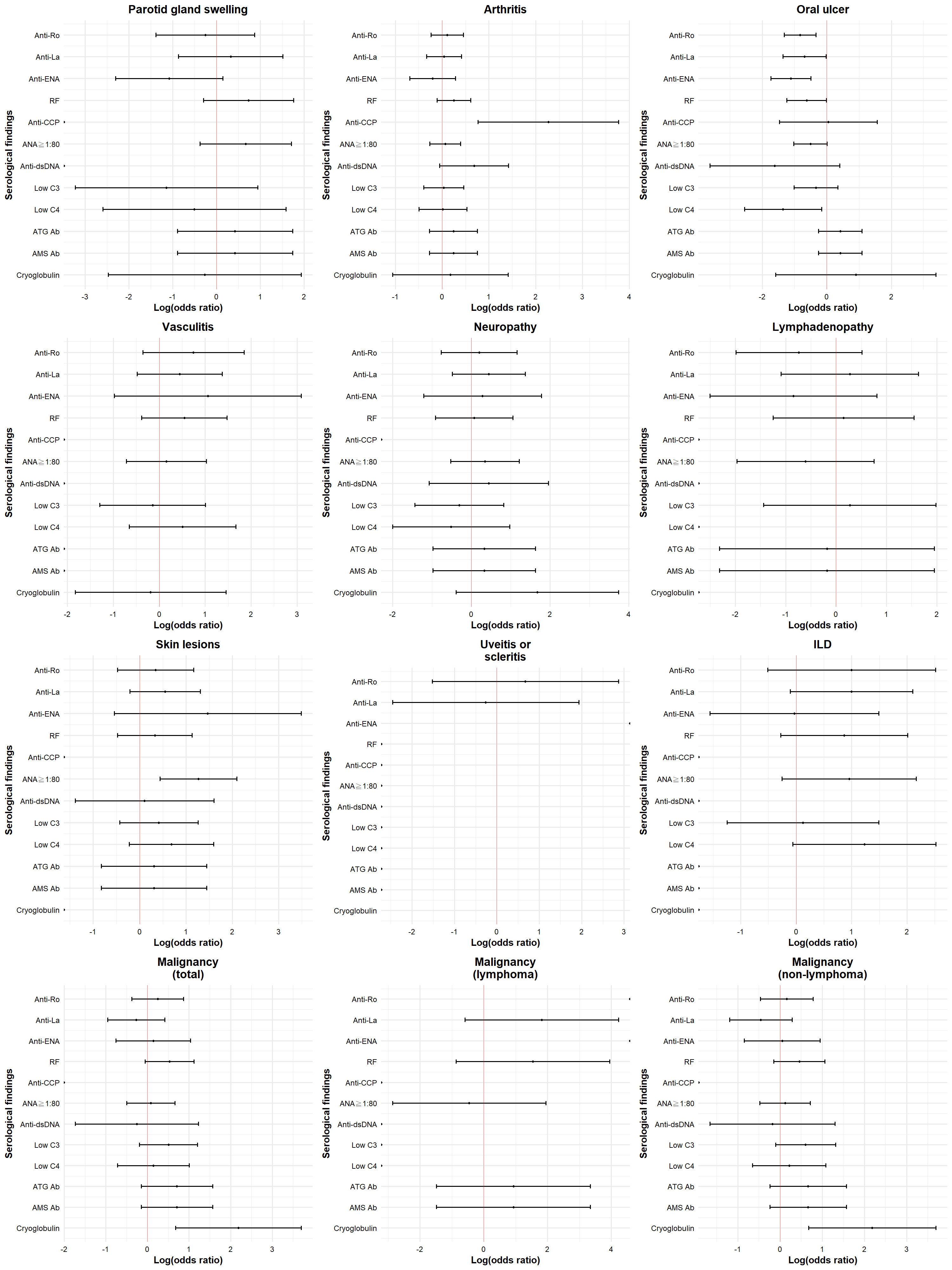


Figure S6. Age at disease diagnosis by HLA-DR genotype


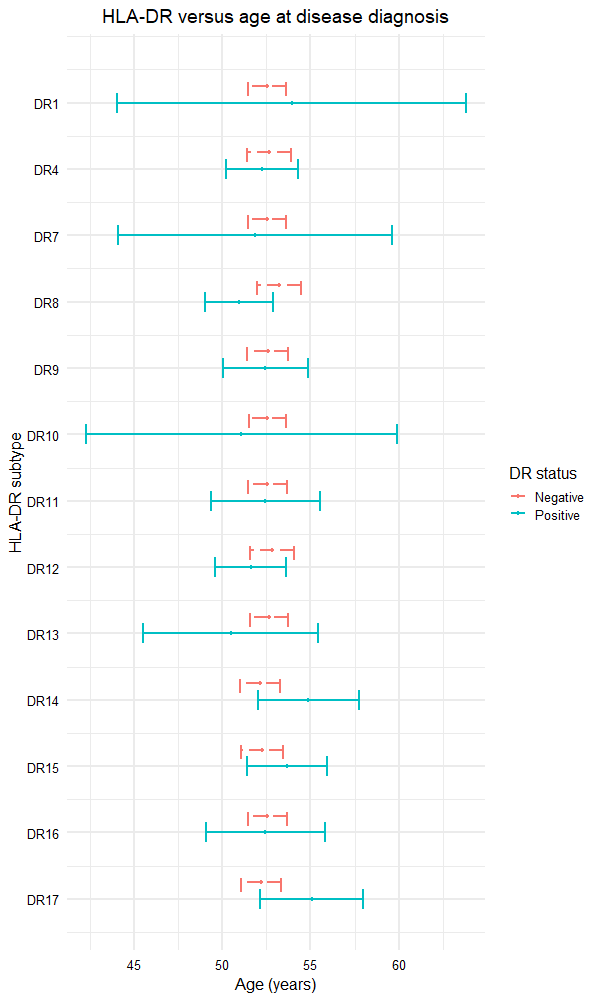

Supplement: Supplementary file 1 — Data S1. Supporting information. [file KJM2-40-934-s001.docx]
